# How obstetricians experience stillbirth and perinatal loss: a systematic review and meta-synthesis

**DOI:** 10.1016/j.xagr.2025.100465

**Published:** 2025-02-16

**Authors:** Frances Cates, Sara Wetzler, Tabitha Wishlade, Mehali Patel, Catherine E. Aiken

**Affiliations:** 1 Department of Liberal Arts (Cates), University of Texas at Austin, Austin, Texas; 2 Department of History and Philosophy of Science (Wetzler), University of Cambridge, Free School Lane, Cambridge, United Kingdom; 3 Department of Obstetrics, Gynecology, and Reproductive Science, Icahn School of Medicine at Mount Sinai (Wetzler), New York, New York; 4 Department of Obstetrics and Gynaecology (Wishlade and Aiken), University of Cambridge, The Rosie Hospital and NIHR Cambridge Biomedical Research Centre, Cambridge, United Kingdom; 5 Sands, the Stillbirth and Neonatal Death Charity (Patel), London, United Kingdom

**Keywords:** high-risk pregnancy, maternal-fetal medicine, obstetrician experience, perinatal loss, qualitative study, stillbirth, systematic review

## Abstract

**Objective:**

Globally, ∼2 million babies are stillborn annually, many in low- and middle-income countries. We aim to understand the experience of obstetricians caring for parents who experience stillbirth and perinatal loss across global settings.

**Data Sources:**

*:* Medline via Ovid, Embase via Ovid, CINAHL via Ebsco, PsychINFO via Ebsco, Scopus, Web of Science Core Collection, and ASSIA via Proquest were searched, database inception-June 2024.

**Study Eligibility Criteria:**

Studies with qualitative components describing experiences of obstetricians providing stillbirth care in any global setting.

**Study Appraisal and Synthesis Methods:**

The Critical Appraisal Skills Programme checklist for qualitative research was utilized to conduct quality assessment. NVivo software was employed for inductive coding and thematic analysis.

**Results:**

Thirteen qualitative studies from both low- and high-resource settings met the inclusion criteria for meta-synthesis. We identified several major themes including the emotional burdens experienced by obstetricians providing stillbirth care, the challenges of patient-provider interactions following adverse outcomes, and a lack of support and resources. Obstetricians across global settings felt devastation, guilt, blame, and a sense of personal responsibility following stillbirth. Obstetricians struggled to navigate the burden of expectation placed on their overall provision of care and tended to question their own professional competence. A subset of obstetricians felt unprepared for the complexity of patient-provider interactions following stillbirth.

**Conclusions:**

Obstetricians experienced complex and conflicting emotions, citing high emotional burden from managing stillbirth cases. Obstetricians identified lack of training and support for providing bereavement care across healthcare settings, indicating a gap that should be filled by stillbirth and bereavement care interventions and education in obstetrical training programs across global settings. Infrastructure for bereavement care training and support systems for obstetricians are crucial to improve the quality of stillbirth and perinatal loss care and prevent an exodus of needed providers for women's care worldwide.

**AJOG Global Reports at a Glance**

**Why was this study conducted?**

• This study was conducted to explore the experiences of obstetricians in cross cultural contexts managing stillbirth and perinatal loss.

**What are the key findings?**

• Obstetricians across settings experience emotional burdens in the form of blame, guilt, and powerlessness following stillbirth or perinatal loss.
• Obstetricians in high- and low-resource settings navigate complex patient interactions and lack bereavement care training or support for coping with adverse events.

**What does this study add to what is already known?**

• Peer-support for obstetricians following traumatic outcomes could improve their personal wellbeing and professional longevity
• Obstetricians across different global contexts identified that more bereavement care training would enhance the quality of care they provide to families.

## Introduction

An estimated 2 million babies per year are stillborn, yielding a global stillbirth rate of 13.9 per 1000 births.^1^ Stillbirth disproportionately affects families in low- and middle-income countries (LMIC), with 98% of stillbirths occurring in LMIC settings.^2^ Due to the high stillbirth burden in LMIC, it is crucial to develop care provision and support strategies for parents and healthcare professionals that can be adapted to any global setting.

Even in higher income countries, obstetricians consider stillbirth to be one of the most traumatic clinical outcomes they encounter,^3^, ^4^, ^5^, ^6^ with most obstetricians reporting experiences of grief following provision of stillbirth care.^7^ In a survey of American obstetricians, the majority reported that stillbirth exerted a large emotional toll, and almost 1 in 10 obstetricians considered leaving obstetric practice after managing stillbirth cases.^8^ Obstetricians report feelings of loneliness and emotional vulnerability associated with stillbirth events ^5^ and cite feelings of helplessness, often related to systemic factors like lack of training, understaffing, and high workload.^9^^,^^10^ Although demographic characteristics of obstetricians do not appear to influence their level of distress following perinatal death,^11^ factors such as high patient turnover, cumulative losses experienced, and lack of support may contribute to greater psychological and emotional impacts.^7^^,^^11^

The experiences of bereaved parents, midwives, and nurses are well-described in the literature^7^^,^^12^^,^^13^ [35440427], however, much less is known about the experiences of obstetricians. To improve stillbirth care, it is necessary to explore the experiences of obstetricians, in particular to identify areas where additional infrastructural and institutional support may be necessary. We therefore aimed to systematically review and meta-synthesize qualitative reports of obstetricians’ experiences of providing care in cases of stillbirth and perinatal loss.

## Methods

### Search strategy

This systematic review adhered to the Prisma guidelines. The protocol for the review was prospectively registered with the international prospective register of systematic reviews (PROSPERO: CRD42024533371). We searched 7 databases (Medline via Ovid, Embase via Ovid, CINAHL via Ebsco, PsychINFO via Ebsco, Scopus, Web of Science Core Collection, and ASSIA via Proquest) on April 7, 2024. We initially used broad search criteria (Supplementary Appendix A) to identify obstetric experiences with stillbirth and perinatal loss. We also performed a manual search of Google Scholar and the reference lists of included studies to ensure that our search was comprehensive.

### Selection criteria

All qualitative or mixed-methods (with qualitative findings reported) research in peer-reviewed journals were considered against our prespecified eligibility criteria. We included any study containing qualitative interviews with obstetricians who provided care to families who experienced stillbirth or early neonatal death. It is important to note that the majority of studies did not distinguish between intrapartum and antenatal stillbirth, while two studies focused solely on intrapartum death.^15^^,^^16^ Studies not reporting qualitative data, books or book chapters, theses, dissertations, opinion pieces, meeting abstracts were all excluded. We also restricted our inclusion criteria to studies in English only.

Studies that described doctors who acted as decision-making obstetricians in their own setting were selected for inclusion. We did not place limitations on the specific training and experience of doctors to ensure a cross-context approach. Where there were mixed populations of training and independently-practicing obstetricians, we included both perspectives where the authors stated that their reports of their experiences did not differ substantively.^3^^,^^14^^,^^15^ We included experiences of both trainees and independently-practicing obstetricians only when it was explicitly stated that their experiences managing stillbirth care were not significantly different; otherwise, we removed trainees from our analysis due to differences in care provision experiences and in assignment of blame between trainees and independently-practicing obstetricians. We did not include studies that reported miscarriage care or later neonatal deaths; however definitions do vary across global settings, therefore we included all studies where losses were defined as stillbirth or early neonatal loss according to local criteria. High- and low-resource settings were characterized based on income, with low-resource settings indicating low- and middle-income countries (LMIC).

### Assessment of risk bias

The quality of the included studies was assessed using the Critical Appraisal Skills Program to reduce risk of bias.

### Data synthesis

At least two authors independently reviewed all studies for inclusion, with a third reviewer available to adjudicate disagreements. After familiarization with the studies, one author completed initial coding using an inductive approach. NVivo v.14 software was utilized for data extraction to code first-order (participant quotations) data. Following initial coding, two other authors reviewed the codes. Discussion with the three authors resolved disagreements. After finalizing the codes, the authors employed inductive thematic analysis to develop themes and subthemes describing the experiences of obstetricians with stillbirth and perinatal loss. Codes were categorized based on this thematic framework.

## Results

### Study selection

Our search yielded 18,052 studies with 10,029 unique studies remaining for screening after removing duplicates. The final thematic synthesis included 13 qualitative studies reporting on the experiences of 127 obstetricians (Table).

**Table:** Summary of studies

Study	Country	No. of participants	Population	Study focus	Data collection	Data analysis	Summary of themes
Choummanivong et al.^17^	Lao People's Democratic Republic	n=33 overall, 7 of whom were doctors/obstetricians	Healthcare professionals who provided care for stillbirth	To understand healthcare professionals’ experiences providing stillbirth care in context of the Lao PDR healthcare system	Semi-structured interviews	Thematic analysis	Lack of auditing/review of stillbirth, lack of support infrastructure for mothers and healthcare professional experiencing stillbirth, limited care provision resources, and lack of staff training contribute to poor stillbirth care management and experiences. Variations in knowledge of stillbirth across and expectation that healthcare providers should know what to say and do in stillbirth cases. Lack of guidelines for stillbirth and bereavement care.
Hendriks & Abraham (2022)^50^	Switzerland	n=10 overall, 1 of whom was an obstetrician	Healthcare professionals who managed stillbirth care	To investigate parents’ views on perinatal loss and to understand the decision-making and support strategies provided by those who cared for them.	Semi-structured interviews	Content analysis	Need for improved decision-making support and empathetic care for parents undergoing termination of pregnancy. Healthcare professionals are challenged to balance the interests of mother and baby while remaining cognizant of the mothers needs/desired and legal regulations. High level of personal responsibility on obstetricians in termination of pregnancy cases, both emotional and legal. Lack of continuity care and fragmented flow of information between providers and parents influences poor care experiences. Lack of standardized bereavement services.
Kelley & Trinidad^19^	United States	n=22 overall, 8 of whom were OB-GYNs	OB-GYNs involved in stillbirth (n=14)	To understand parents’ experiences with stillbirth in light of care provided by OB-GYNs and to investigate OB-GYNs’ intentions and efforts to support parents who experience stillbirth	Semi-structured focus groups	Thematic analysis	Stigma and taboo around stillbirth contribute to isolated suffering of parents and difficult interventions by healthcare providers. Grief of parents not recognized by healthcare providers or societally. Variation in knowledge of stillbirth across providers. Balance between finding answers and responsibility to provide care following stillbirth, since providers view their role as finding answers and understanding what happened. Need for stillbirth knowledge to training among providers due to their discomfort discussing the topic with patients.
Lappeman & Swartz^14^	South Africa	n=8 medical doctors	Doctors who worked in the labor ward for at least 4 months and experienced at least 1 stillbirth case	To explore doctors’ responses to and coping strategies for stillbirth in the context of a low-resource setting	Free association narrative interview method (FANI) interviews	FANI-guided analysis	Traumas in low-resource, impoverished community drive doctors to employ defense mechanisms against emotions to avoid pain and anxiety; doctors remain emotionally detached. Need for institutional and external support to cope with significant emotional burden. Frustrations with systemic factors such as cultural differences and communication barriers cause disconnect with patients/community. Tendency of doctors to make decisions for patients. Doctors experience overwhelming fear that they have missed something.
Lawrence et al.^24^	Ghana	n=52 overall, 3 of whom were obstetric consultants and 8 of whom were specialists	Obstetric providers at all levels of training and practice	To investigate obstetric providers’ view on seeking mental health help following adverse clinical outcomes, with a focus on the high rates of maternal and neonatal death in this setting.	Focus group interviews	Thematic analysis	Cultural norm to repress emotions after traumatic clinical encounters generates stigma around seeking mental health care. Adverse outcomes compounded by systemic factors, including increased patient volumes, low resources, and poor infrastructural support. Providers experience pressure to move forward after poor outcomes, particularly in limited-resource setting where providers are exposed to high rates of neonatal death. Peer support and debriefing space needed to destigmatize sharing of personal experiences. Double standard that mental health help is acceptable for patients but not practitioners contributes to emotional restraint from providers. Lack of time and training to process poor clinical outcomes.
McNamara et al.^16^	Ireland	n=89 overall, 11 of whom were obstetric consultants	All consultants in the labor ward and NCHDs who worked in the ward between 2010 and 2015	To examine the experiences of healthcare providers exposed to perinatal death and to understand the influence of exposure on future clinical practice, with a focus on education and support efforts for healthcare providers	Questionnaire with combination of open- and close-ended questions	Thematic analysis	Need for peer support and nonjudgmental space to debrief following intrapartum death. High expectations placed on doctors to continue working after trauma; emotional burden is overshadowed by the need to continue providing care. Providers experienced doubts/questioning of their clinical skills.
McNamara, Meaney, O'Donoghue^51^	Ireland	n=10 obstetricians	Obstetricians who worked in large tertiary maternal hospital and experienced direct involvement with intrapartum fetal death (IPD)	To investigate the impact of IPD on obstetricians, with a focus on their attitudes and responses.	Semi-structured interviews	Interpretative phenomenological analysis	Obstetricians experience negative emotional impacts following IPD, including devastation, shock, and sadness. Self-blame and guilt contribute to sense of failure and doubt in clinical abilities and standard of care provided. Culture of blame amplifies need for peer support and opportunities for debriefing. Lack of protection and institutional support, particularly regarding media allegations. Obstetricians place patients’ needs and the need to provide high-quality care above their own emotions and concerns.
Montero, Sanchez, Montoro, Crespo, Jaen, Tirado (2011)^52^	Spain	n=19 overall, 2 of whom were obstetricians	Health professionals in the maternal-infant area who witnessed case(s) of perinatal loss	To explore the experiences and care provision strategies of healthcare providers managing care for perinatal loss	Semi-structured interviews	Phenomenological analysis	Feelings of frustration and impotence fuel providers’ doubts in professional competence and create sense of guilt and failure following perinatal loss. Providers concentrate on physical instead of emotional aspect of care; problems with emotional care are compounded by inadequate skills and resources to provide bereavement care. Providers experience the weight/pressure to deliver bad news to parents and anticipate that parents will blame them. Not knowing what to say or do shows need for education for perinatal loss management.
Nuzum et al.^18^	Ireland	n=8 consultant OB-GYNs	OB-GYN consultants on permanent staff who provided care for stillbirth in a tertiary university hospital in Ireland	To understand OB-GYNs’ experiences and the personal and professional effects of providing stillbirth care.	Semi-structured interviews	Interpretative phenomenological analysis	High expectations placed on providers to facilitate pregnancy/birth places emotional burden on providers. Unrealistic standard that providers are infallible generates sense among providers that they cannot miss anything and increases weight of responsibility on proviers. Need for perinatal bereavement care training due to providers’ discomfort in this area. Legal concerns due to pervasive blame culture. Lack of support structure for providers due to draining nature of providing empathetic care to stillbirth patients.
Petrites et al.^20^	Ghana	N=36 overall, 13 of whom were OB-GYNs	Healthcare providers at a large teaching hospital in Ghana with experiences in perinatal loss	To understand healthcare providers’ experience and strategies’ for coping with high rates of perinatal death, with a focus on how providers make sense of perinatal death.	Semi-structured interviews	Content analysis	Providers demonstrate dedication to pushing through adverse outcomes to search for answers and to provide high-quality care to future patients. Providers’ frustration with systemic and external factors for contributing to preventable perinatal deaths. Emotional distance and detachment of providers compounded by lack of time and resources.
Power, Meaney, O'Donoghue (2021b)^53^	Ireland	n=10 fetal medicine specialists	Currently practicing fetal medicine specialists	To investigate fetal medicine specialists’ experience providing care for pregnancies complicate by fatal fetal anomaly, in context of Ireland's implementation of termination of pregnancy.	Semi-structured interviews	Thematic analysis	Fear of litigation amplified by concerns that diagnosis was incorrect or not fatal enough, in light of new legislation allowing termination of pregnancy (TOP). Feelings of isolation, vulnerability, and internal conflict due to TOP provision; emotional detachment used to cope with brutal experience of providing TOP. Increased workload combined with lack of institutional and peer support adds burden to providers.
Serafim et al.^21^	Brazil	n=11 overall, 3 of whom were obstetricians	Professionals working in women's health care and obstetric care	To explore healthcare professionals experiences with intrauterine fetal death, with a focus on the lack of support and training for professionals providing IFD care.	Semi-structured interviews	Thematic analysis	Defensive, detached attitudes demonstrated by healthcare providers to protect against and avoid emotions due to lack of support for coping with emotions following intrauterine fetal death. Need for support network and space for providers to share experiences. Communication challenges with parents amplified by lack of training and preparation; role of obstetricians as first point of contact when breaking bad news. Obstetricians torn between following protocols/providing the necessary care to patients and treating them with empathy. No significant difference in responses between trainees and consultants.
Sheen, Goodfellow, Balling, Rymer, Weeks, Spiby, Slade^54^	United Kingdom	n=43 consultant obstetricians, trainees, and other Royal College of Obstetricians and Gynaecologists	RCOG members who experienced work-related trauma and consented to interview	To understand obstetric events experienced as most traumatic by RCOG members and to identify factors contributing to their traumatic nature	Semi-structured interviews	Content analysis	Events identified as traumatic were similar between consultants, trainees, and other RCOG members; most difficult events were maternal or neonatal death, hemorrhage, or difficult delivery. Traumatic nature of events compounded by unpredictability, preventability, and high emotionality. Need for training around traumatic events and development of support strategies. Sense of responsibility and self-blame contribute to feelings of isolation, professional helplessness, and impotence.

### Study characteristics

Studies were conducted in 9 countries (, Brazil, Ghana, Ireland, Lao People's Democratic Republic, South Africa, Spain, Switzerland, United Kingdom, United States). The included studies utilized various research methods, including semi-structured interviews and focus group discussions and employed qualitative analysis methods such as thematic analysis, content analysis, free association narrative interview (FANI)-guided analysis, and interpretative phenomenological analysis.

### Risk of bias of included studies

Based on the CASP 18 evaluation, the studies were generally of good quality. However, some studies did not adequately describe the relationship between participants and researchers (Supplementary Table 1). Other studies did not provide clear statements of findings.

## Synthesis of results

### Emotional burden experienced by obstetricians

Emotions commonly experienced by obstetricians providing stillbirth care included devastation, sadness, grief, blame, guilt, and feelings of isolation. Obstetricians emphasized the immense emotional weight exerted by stillbirth and perinatal loss, and expressed a strong sense of personal responsibility. There was little institutional recognition of the emotional burdens obstetricians face and most obstetricians reported lacking opportunities for support. Many obstetricians felt isolated and left alone to manage their grief and devastation. Obstetricians also expressed frustration at institutional expectations placed on doctors to cope with traumatic outcomes on a regular basis.

“An unexpected intrapartum event can be devastating. In the few cases where I have been directly involved I have asked the affected doctor to go home. It is akin to a train driver witnessing a suicide jump or crashing a car into a pedestrian. We would never expect people to work normally after these events so why should doctors?” ^16^ (Ireland)

Obstetricians in both high- and low-resource settings experienced the emotional burden of stillbirth. However, factors contributing to the emotional toll were context-dependent. In low-resource settings, obstetricians described how the inability to provide high quality care added to the emotional weight of stillbirth experience, whereas in high-resource settings obstetricians were more likely to voice fears of litigation or public blame following stillbirth that increased their emotional baggage.

I. Guilt and blame

Obstetricians very often referred to concepts of guilt and blame, and expressed feeling at fault for stillbirth, even when they also felt there was nothing they would have done differently. Particularly in low-income settings, obstetricians experienced guilt when they could not provide the needed quality of care due to systemic factors or when they believed deaths were preventable. Additionally, guilt and blame can be linked to feelings of failure.

“I have an awful lot of guilt but it is probably irrational guilt... .it is coming back to the what ifs but you often say to yourself even though you have no clinical indication what if and they would have been this perfect happy family” ^15^ (Ireland)

“To this day I don't know if I could have done anything differently and I don't know if I anything I did caused this but at the time.., you know it must be my fault.” ^15^ (Ireland)

“We induced the mother into labour, she was pushing, but by the time the baby was delivered it was too late, the baby was dead. It was a stillbirth. I was frustrated with the system. Why was I frustrated with our poor health system? If we had the capacity to perform caesarean section, the baby could possibly have survived. I felt guilty and blamed myself, because the baby had a beating heart when the mother presented. This will haunt me. I was not able to save this human.” ^17^ (Lao PDR)

Across settings, obstetricians struggled with the question of preventability, describing how preventable deaths haunted or followed them.

II. Feelings of impotence and powerlessness

Obstetricians described feelings of powerlessness when dealing with perinatal loss and discussed how they felt helpless when the procedures and protocols they were trained to perform did not prevent losses. Some obstetricians were able to cope with their perceived powerlessness in certain cases and recognized that they did everything they could for patients, while others experienced a vicious cycle of self-blame, particularly in high-resource settings. In low-resource settings, obstetricians’ recognition and acceptance of systemic factors impeding their ability to provide care may have alleviated some self-blame, since blame could be attributed to factors outside their control.

“I think it was that it felt as though the baby was going to die in front of me, that I was powerless, I was doing my very best to rectify the situation but it was sort of that feeling that the baby is going to die right in front of my eyes, this baby is going to die and I can't seem to do anything about it...” ^3^ (United Kingdom)

III. Weight of responsibility and expectation to be infallible

Obstetricians felt intense pressure to avoid mistakes. Particularly in high-resource settings, obstetricians expressed feelings of isolation due to the burden of expectation on their performance, in particular as the final clinical decision-makers. Contributing to the weight of responsibility was the impending dread of delivering bad news to parents.

“As a fetal medicine specialist you're not allowed to miss anything which is completely nonsense, but you don't allow yourself to miss anyone (mother/baby).” ^18^ (Ireland)

“The hardest part now is that your name is at the top of the chart and when it all goes wrong even if you're not involved ... it all comes back to the consultant ... you're ultimately responsible.” ^18^ (Ireland)

“As doctors you have these moments from time to time where you are solely responsible for that thing and nobody else can help you out, there's only you. And those moments can be pretty, pretty lonely… and pretty terrifying.” ^3^ (United Kingdom)

Obstetricians grasped the lifelong impact of perinatal loss on parents and feared the impact bad news would have on families and communities, particularly in low-resource settings.

“People…are not changing their attitude because they think that, ‘Oh, this is just one person dying…’ But if you see the gravity of the issue, …it might not be a thousand, but you get somebody to understand that even that one death…could send a whole family going down because the leader of that family has gone mental, and that means father, mother, grandfather, cousins, somebody, who you are supporting…have lost their support. So, it's not just about some baby who hasn't got a name dying, that's, there's wide and diverse complications and implications. And when people come to comprehend that, attitudes, I'm sure, will change.” ^20^ (Ghana)

In high-resource settings, some obstetricians felt that societal expectations regarding pregnancy outcomes did not align with reality, for example that there are risks inherent in some pregnancies. The burden of parental expectations weighed heavily on obstetricians when adverse outcomes occurred. Further exploration is needed to understand how the social status of obstetricians influences expectations and obstetricians’ feelings of guilt, failure, and blame when expectations are not met.

“We have created an expectation that nothing can go wrong.” ^15^ (Ireland)

Doctors acutely feel the expectations patients have for a healthy pregnancy, though further research is needed to understand whether obstetricians sufficiently discuss the risk for stillbirth with expectant parents. It is possible that these conversations, while difficult, might lessen the pressure of expectations expressed by obstetricians.

IV. Searching for answers or second-guessing professional competence

Many obstetricians attempted to process adverse outcomes by seeking to understand the causes underlying cases of perinatal loss and stillbirth. Especially in high-resource settings, obstetricians commonly second-guessed their clinical decision-making and competence, and this was often highly correlated with self-blame and guilt. This tendency was less common in low-resource settings, perhaps due to obstetricians’ acceptance of systemic factors hindering their care.

“It's hard. Sometimes once you have done the workup and told them you can't find any reason, or this is the reason and this what you are going to need next time, I think sometimes you still can't give them everything they want.” ^19^ (United States)

“You are always in the back of your mind thinking what could I have done better? What did I miss? If I had gotten her to theater two minutes earlier would the neonatal team have been able to save the baby?” ^15^ (Ireland)

In low-resource settings, obstetricians cited improving care for future patients as their motivation to search for answers behind adverse outcomes, while in high-resource settings, obstetricians sought answers so they could better inform families of what happened. Obstetricians in high-resource settings were also motivated to find answers to protect themselves from blame and potentially legal consequences.

V. Pressure to move forward following adverse outcome

Along with the pressure to perform, obstetricians experienced pressure to continue with their work following perinatal loss and stillbirth. Especially in low-income settings with limited resources, obstetricians felt pushed to move on from traumatic outcomes, with little time for support or debriefing.

“After (an infant death) I felt actually traumatised and I sort of avoided going to the labour ward for a while. But you have to get back into it because there's no other option.” ^14^ (South Africa)

“When you come in and you see people in need, people suffering, you have no choice but to go on and on and on. Nothing special…it's just the, zeal and the will to do what is good.” ^20^ (Ghana)

While pressure was experienced across all settings and support was lacking, obstetricians in some societies acutely felt pressure due to cultural norms that stigmatized sharing experiences, even with peers and colleagues. While obstetricians in some cultures felt relieved of pressure after sharing with peers, others lacked an outlet for support and discussion due to stigma.

### Provider-patient dynamics

Obstetricians discussed the challenges of providing quality clinical stillbirth care while also emotionally supporting bereaved parents. Obstetricians’ search for the causes behind adverse outcomes conflicted at times with parental desire for emotional care and empathy. The struggle to balance clinical care with emotional support was experienced by obstetricians across all settings. Obstetricians described the challenges of empathetic communication in the wake of highly emotional, traumatic events, especially when delivering bad news for the first time. Some obstetricians cited this as the worst part of their job.

“The hardest part, I think, is the emotional support for us because we have to separate ourselves from that and be the clinician, and I find that is the hardest to do both—be there as their friend and then also step back and figure out why for them.” ^19^ (United States)

“Be with (the mother), identify, put myself in her state, let her understand that I share in her loss, that I share in her sorrow.” ^20^ (Ghana)

“I speak to them the way that I'd like somebody to say to me .. . that's sympathetic.” ^18^ (Ireland)

I. Communicating with families

Obstetricians placed priority on the families’ feelings, trying to set aside their own emotions to provide the best possible support. Obstetricians showed recognition of the devastation experienced by families and tried to employ strategies sensitive to their needs, in particular trying to “humanize” interactions. Several obstetricians believed that parents would likely not remember what they said, but instead how the conversation made them feel.

“I try to put the pregnant woman and her family in the room with privacy; use objective language appropriate to their level of understanding. One of the strategies is to check if the pregnant woman, family members are prepared to receive the news and periodically evaluate the understanding of what was said, acting with empathy for the reactions that may arise.” ^21^ (Brazil)

“I guess you compare the situations you've been in ... and how they would feel and what they would like to hear and often they don't want to hear the clinical stuff they just want somebody human ... they remember for the rest of their life a few people associated with that. It's more your expression, your body language and ... how you are with them.” ^18^ (Ireland)

“The priority has to be the parents and talking to them at this stage is hugely important ... no matter how much you are worried about yourself you have got to just rise above that and you have got to go in and empathise.” ^15^ (Ireland)

Though obstetricians across settings reflected on the need for empathetic communication, they discussed the challenges of such communication. Obstetricians never grew accustomed to delivering such devastating news and voiced their dread of this role. In times of high grief, obstetricians may experience a gap in sensitive, empathic communication with patients. Further investigation is needed to understand the patient perspective on communication with obstetricians after stillbirth, as the patient perspective may elucidate the level of empathic communication that providers offer during interactions following stillbirth

“You receive the first impact, because you're the one who has to give the bad news, you don't get accustomed to that and, hence, the way of giving the news depends on each professional's individuality.” ^22^ (Spain)

“How in the hell am I going to tell this woman who has just had a general anesthetic, who is asleep, how in the hell am I going to wake her up and tell her that her baby is dead?” ^15^ (Ireland)

II. Impact of fear of litigation on patient-provider interactions

In high-resource settings, obstetricians expressed an intense fear of litigation. Obstetricians were extremely wary of legal consequences following adverse outcomes and altered their communication strategies to avoid missteps with families. Obstetricians demonstrated frustration with blame culture and media scrutiny. Fear of litigation amplified the weight of responsibility and emotional burden obstetricians experienced and led them to carefully tailor interactions.

“Every patient reacts differently, and you don't know at that moment which patient this is. Is it the one that is going to blame you? Is it the one that's going to want your comfort? And so you are caught. Probably because of malpractice, it's like, you know, “How am I going to approach this patient?” With guarded emotions. You know? If I am close to the person, then I am comforting.” ^19^ (United States)

“People are nervous about being that first person who might be prosecuted... when people have to put their name on something or their head is on the line there is a fear for sure.” ^23^ (Ireland)

“Patients are more litigious than they used to be and most of them there isn't a case but that destroys our lives for about 3 or 4 months you know.” ^3^ (United Kingdom)

III. Changing relationships with families

The transition from pregnancy care to bereavement care proved a challenge due to the significant relationship change with parents. Across healthcare settings, obstetricians felt unprepared to provide bereavement support and commonly expressed the desire to transition stillbirth cases to grief counselors or psychology services whom they felt were better equipped.

“(With stillbirth) you transition from being a doctor to a hand holder, a grief counselor. Compared to prematurity—you know you have a baby; you've got a premature baby. You become hopeful, kind of a cheerleader along the way. The team transitions too, because as an obstetrician once pregnancy ends, while you are still involved, that baby gets taken over by the pediatrician or pediatric team. I won't say the relationship ends, but it definitely changes. Whereas with stillbirths there is no transition of care, and you are kind of left being that counselor.” ^19^ (United States)

Some obstetricians in high- and low-resource settings described emotional detachment from and avoidance of parents following stillbirth. While some obstetricians failed to provide emotional support due to time and resource constraints, others avoided emotional care because it evoked their own emotions. These obstetricians preferred to remain emotionally detached to protect their own mental health and to shield themselves.

“I do see them as a diagnosis … I'm not a huge fan of talking about personal things, so I tend to not check in with the patients on an emotional level. I'm kind of like, I have questions that I want to ask, answer them, and then I'm walking away kind of thing.” ^14^ (South Africa)

“But I do think that a big part of it is like an unconscious coping, like try and separate yourself or remove yourself from the community so that you are less affected about what happens to people. ^14^ (South Africa)

“Many parents complain about the solitude they experience in these situations, the fact is that the professionals react by moving away.” ^20^ (Ghana)

“In this part of the world. in Ghana land, we try to put on tough skin with most of the situations we, we find ourselves in… (we) muzzle most of the things in… we don't expect so much of your emotions.” ^24^ (Ghana)

### Lack of stillbirth or bereavement care training

Obstetricians across healthcare settings expressed their need for more stillbirth and bereavement care training. Obstetricians felt overwhelmed by the complexities of bereavement care when there were no standard operating procedures.

“The team is not prepared at all ... Not only in terms of training, but also in terms of the institution: there is no work in relation to this. I think that even awareness raising does not exist.” ^21^ (Brazil)

“After a stillbirth, as the senior doctor I am expected to explain to the mother and family what happened. I understand that mothers need to be supported from the time the baby was suspected to be dead, but the majority of the time the mother does not receive that right care. In (LMIC), we have no bereavement care. So not all our staff are familiar with the right care.” ^17^ (Lao PDR)

“...often you find people feel out of their depth” ^18^ (Ireland)

“So it's difficult and you can't just go through that tick-box sheet. I even feel uncomfortable talking to them about it, then I don't … I just kind of have to say that I'm sorry for the loss, and then that's as far as I will go, because distancing myself from the problem is better than overstepping something and causing worse emotions. Like I'm causing more emotional trauma.” ^14^ (South Africa)

I. Lack of support and spaces for sharing experiences

In both low- and high-resource settings, obstetricians voiced their frustration with the lack of time or space for debriefing their own experiences of adverse outcomes.

“There was no recognition that it might be difficult ... there was no training ... there was no debriefing ... and I think that's bad. You did it yourself ... nobody cared if you got so psychiatrically disturbed you threw yourself off the roof the following week.” ^18^ (Ireland)

“There's no formal debriefing for anywhere in this hospital, for any specialty, and it is something that we have raised before, saying that there should be … a lot of health practitioners are suffering from mental health because of it.” ^14^ (South Africa)

“There is nothing. I have worked in seven hospitals, seven maternity units and I have never been formally debriefed, after a stillbirth or after an intrapartum death... I don't even think people asked us were we ok after it, we just continued on working and that was it you know.” ^15^ (Ireland)

In this vein, obstetricians in high- and low-income settings expressed a desire for spaces to share their experiences with peers. Informal support from colleagues was cited as a common coping strategy for dealing with stillbirth

“It is necessary to talk more about this in the work environment... The worker needs protected spaces in his workload to talk about it. And we always need to talk because it shouldn't be a taboo or a veiled subject.” ^21^ (Brazil)

“The best support mechanism is to go to another colleague... going to a professional person who may be very well qualified and very well skilled to do the counseling around it wouldn't work for me as talking to someone who knows exactly what you are going through because it is such an awful lonely emotional journey... I would certainly struggle to articulate just how fucking awful it is.” ^16^ (Ireland)

## Discussion

### Main findings

The impacts of stillbirth and perinatal loss on obstetricians are understudied and underappreciated across healthcare systems. We outline the current understanding of obstetrician perspectives on managing stillbirth, the complex relationship dynamics with families following stillbirths, and the state of stillbirth and bereavement care training and support for obstetricians. We identify opportunities to improve infrastructure supporting stillbirth and bereavement care in a range of healthcare settings.

### Comparison with existing literature

The sense of personal responsibility and weight of expectation amplify the emotional toll on obstetricians in all settings we were able to include in the review. Guilt and blame were universal themes and echoed the emotions experienced by midwives, nurses, and families themselves.^25^, ^26^, ^27^

Obstetricians in all settings felt a lack of support, training, and resources when providing bereavement care, and most obstetricians felt unprepared when their relationship with families inevitably changed after a stillbirth occurred. In low-resource settings, systemic factors exacerbated inadequacies in bereavement care. Patient interactions following adverse outcomes posed a challenge to obstetricians in both high- and low-resource settings, with communication of bad news cited universally as the worst part of obstetricians’ jobs. Obstetricians also struggled under the weight of the expectation to be infallible; similar challenges with perfectionism and infallibility have been reported in other medical fields, including pediatrics.^28^^,^^29^ Obstetricians in all settings used emotional detachment as a coping strategy for managing such cases, with many obstetricians highlighting the emotional toll of providing empathetic care. The tendency to separate emotions from clinical care may stem from compassion fatigue and burnout, which are common in obstetricians.^30^, ^31^, ^32^, ^33^ Navigating the balance of protecting obstetricians’ emotional well-being while also responding to family needs requires further awareness, training and infrastructural support from specialist bereavement teams to provide empathetic, parent-centered care.^34^, ^35^, ^36^ In future training, it is vital to address communication strategies for delivering bad news, so that providers can feel confident in their ability to thoughtfully and compassionately communicate with families while also minimizing the risk of litigation.^37^^,^^38^

Many healthcare systems lack robust infrastructure for bereavement care. In the low-resource settings included in our study, obstetricians described how there was often no bereavement care whatsoever. A critical research goal will be to develop a training and care provision framework for stillbirth that can be adapted around different cultural contexts and applied in low resource settings. Even in high-resource settings, many obstetricians discussed the lack of bereavement care frameworks that they could easily access. Previous research reflects the dearth of perinatal death training for healthcare professionals,^39^, ^40^, ^41^ suggesting that failures to provide stillbirth care training extend as far as the early stages of obstetric education. Especially given the common misperception that obstetrics is a perennially positive field, it is crucial to provide trainees with simulations and other application-based tools that allow them to practice “worst-case” scenarios. Overall, training should equip obstetricians across settings with the tools to effectively coordinate poststillbirth care.

Our work highlights that obstetricians lack opportunities and resources for support following stillbirth and perinatal loss. Obstetricians in all settings voiced their desire for more formalized peer support, for example spaces for mentorship and experiential sharing. Peer support groups have shown success in improving well-being, decreasing stigma, reducing burnout, and creating more positive culture.^42^^,^^43^ However, to promote efficacy of peer support groups, it is necessary to implement mechanisms that ensure physician engagement. Promoting engagement in and implementation of obstetrician support could contribute to a more constructive framework around caring for families who experience stillbirth. It is important that these groups are safe and authentic spaces for reflection and vulnerability, supported by structured guidelines.

Examples of initiatives to improve poststillbirth care and provide resources to bereaved parents ^44^^,^^45^ provide useful models, on which initiatives to support obstetricians might be based. There are also good models in different global settings for education programs in bereavement care training and stillbirth management skills, e.g. IMPROVE in Australia ^46^^,^^47^ and the Saving Babies Lives Care Bundle in England, which lays out evidence-based guidelines for stillbirth prevention.^48^ Furthermore, the recent Lancet Stillbirth series ^49^ issues a call to action for preventing stillbirth and positions supporting both families and care providers as a key focus.

### Strengths and limitations

Strengths of our study include the wide range of cultural contexts encompassed by the articles selected for inclusion, saturation reached upon thematic analysis, and the array of perspectives captured by exemplified quotes. A limitation is that we were unable to differentiate between independently-practicing and training obstetricians in some settings, though including obstetricians at different levels of training provided more perspectives. Furthermore, since the functional definition of an obstetrician varies across cultures, there was heterogeneity with respect to this within the analysis. Although we captured the lack of bereavement care training and support across healthcare settings, further research is needed to determine the best methodology and infrastructure for improving bereavement care and obstetrician support.

### Conclusions and implications

Obstetricians frequently experience devastation and guilt following stillbirth, and subsequent interactions with families can be complicated by these feelings. Failure to implement procedures, training, or resources for stillbirth care provision beginning in medical school contributes to feelings of frustration and isolation in obstetricians. We identify the need for increased support of obstetricians following traumatic outcomes that would improve their personal wellbeing, professional longevity, and the quality of care they provide to families.

## CRediT authorship contribution statement

**Frances Cates:** Writing – original draft, Formal analysis, Data curation, Conceptualization. **Sara Wetzler:** Writing – review & editing, Data curation, Conceptualization. **Tabitha Wishlade:** Writing – review & editing, Formal analysis, Data curation. **Mehali Patel:** Writing – review & editing, Conceptualization. **Catherine E. Aiken:** Writing – original draft, Formal analysis, Data curation, Conceptualization.

## Conflicts of interest

MP is an employee of Sands, a charity whose work includes providing bereavement care training to professionals in the UK.

## Appendix Supplementary materials

Image, application 1

Image, application 2

## Footnotes

Supplementary material associated with this article can be foun, in the online version at doi:10.1016/j.xagr.2025.100465.
