# Supplementary material for: How obstetricians experience stillbirth and perinatal loss: a systematic review and meta-synthesis
Source: AJOG Glob Rep. 2025 Feb 16;5(2):100465. doi: 10.1016/j.xagr.2025.100465 (PMC11931382; doi:10.1016/j.xagr.2025.100465)
Supplement: Supplementary file 1 [file mmc1.docx]

Supplementary Table 1: Critical Appraisal Skills Program Assessments

| **Review**  **1= experience**  **2= hows**  **3= impact of hows** | **Authors** | **Year** | **Was there a clear statement of the aims of the research** | **Is a qualitative methodology appropriate** | **Was the research design appropriate to address the aims of the research** | **Was the recruitment strategy appropriate to the aims of the research** | **Was the data collected in a way that addressed the research issue** | **Has the relationship between researcher and participants been adequately considered** | **Have ethical issues been taken into consideration** | **Was the data analysis sufficiently rigorous** | **Is there a clear statement of findings** | **How valuable is the research** | **Narrative appraisal** |
| --- | --- | --- | --- | --- | --- | --- | --- | --- | --- | --- | --- | --- | --- |
| 1+2 | Choummanivong, M., Karimi, S., Durham, J., Sychareun, V., Flenady, V., Horey, D., Boyle, F. | 2020 | Yes, to understand the experiences and management of stillbirth in the Lao People’s Democratic Republic, a lower-middle-income country. | Yes, semi-structured qualitative interview with health professional to investigate experiences of providing stillbirth care. | Yes, semi-structured interviews of 33 healthcare professionals (n=7 doctors/OBs) were conducted using an interview guide based on work from The Lancet Ending Preventable Stillbirth Series. Researchers asked open-ended questions to draw out views and experiences of participants. | Yes, purposive sampling was conducted based on experience and knowledge of facilities to ensure provision of detailed information. | Yes, interviews were conducted by a researcher with post-graduate training in qualitative public health research and assisted by a Laotian qualitative researcher. Interviews ranging from 25 to 90 minutes occurred in June 2018 and were held at the interviewee’s healthcare facility. Participants worked at the central, provincial, and district hospital level in Lao PDR. | Not sure, it is not specified whether the researchers established prior contact or rapport with participants. | Yes, approval was obtained from the Human Research Ethics Committee and the Lao PDR University of Health Sciences Ethical Review Board. Researchers obtained written informed consent from all participants. | Yes, thematic analysis was conducted using a combination of inductive and deductive techniques. Though one author led analysis, they were supported by regular discussion with other authors. | Yes, it is clear that an increased stillbirth awareness and training among healthcare professionals is needed to improve care experiences. Healthcare professionals lack the training and knowledge to provide adequate care, especially bereavement care. | This research makes an important contribution to the limited research on stillbirth experiences in low-resource settings, since the majority of research is conducted in high-income countries. | Sample size of 7 OBs limits the application of the findings. Further research is needed to understand the experiences of OBs in low-resource settings. Findings demonstrate the lack of bereavement care training and general knowledge of stillbirth care. |
| 1 | Hendriks, M.J., Abraham, A. | 2022 | Yes, to gain insight into the views of parents and healthcare providers involved in the practice of late termination of pregnancy at 20 weeks gestation or greater | Yes, semi-structured interviews with parents and perinatal healthcare providers investigated the experiences of and decision making involved in late termination of pregnancy. | Yes, a semi-structured interview guide was used to conduct interviews with 5 healthcare providers (n=1 obstetrician). HCP interviews took place after parental interviews, so HCPs were first asked to provide commentary on the parental feedback and then to describe their experiences with decision-making regarding late termination of pregnancy. | Yes, purposive sampling of healthcare providers and parents occurred in a Swiss university hospital’s tertiary perinatal center. Parents were invited to participate if they experienced the loss of a child at the perinatal center one or more years before the study. | Yes, interviews with parents ranging from 60 to 160 minutes were conducted in locations selected by the participants (their home or workplace). Interviews with healthcare providers lasting from 60 to 160 minutes also took place in locations selected by the participant (the hospital, home, or other workplace). | Yes, the researchers acknowledged their affiliation with the hospital at which the study was conducted, though in their roles as medical anthropologists they were not involved in medical care. However, it is not specified whether the researchers had established rapport with participants in their capacity as medical anthropologists. | Yes, approval was obtained from the Ethics Committee. Informed consent was provided to participants. Additionally, due to the potential for psychological harm, process consent was obtained throughout the interviews. | Yes, the researchers conducted qualitative content analysis. The researchers developed an initial coding scheme from the interview guide, reviewed literature, and field notes. Subsequently, they independently coded four transcripts to ensure equivalent coding procedures, and once congruence was reached, they divided the remaining transcripts to code using MAXQDA analysis software. | Yes, the researchers emphasized parents’ need for decision-making support when undergoing LTOP. Lack of support was compounded by fragmented care from healthcare providers and communication issues. Additionally, the lack of bereavement care guidelines and services hindered parental support. | The findings contribute to the existing body of knowledge on parents’ experiences with parental loss and highlight parents’ desire for doctors to show emotional involvement in the decision-making process. | The study compares the perspectives and experiences of parents and healthcare providers. While parents express a desire for providers to provide emotional as well as physical care, healthcare providers emphasize the struggle of making the right decisions for patients, especially when legal factors come into play. The complexities of decision-making can impede communication and emotional support provided by HCPs to patients. |
| 1 | Kelley, M., Trinidad, B. | 2012 | Yes, to understand the experiences of parents going through parental loss in light of healthcare providers’ beliefs, intentions, and support efforts. | Yes, semi-structured focus groups investigated the experiences of patients and parents with clinical encounters for stillbirth. Focus groups concentrated on the emotional and personal aspects of parental clinical encounters and sought to understand obstetricians’ beliefs about and views of their patients’ experiences with stillbirth. | Yes, 3 semi-structured focus groups were conducted with parents who experienced a stillbirth, and 2 semi-structured focus groups were conducted with OB-GYNs (n=8). | Yes, parents were recruited from parent hospital guild groups and via the snowball method. OB-GYNs were recruited from the greater Seattle region in Washington state. | No, the length and location of focus groups is not mentioned. Furthermore, it is not clearly mentioned who conducted interviews and whether they were properly trained/qualified. | Not sure, the relationship between patients and providers is not specified. | Yes, approval was obtained from the Seattle Children’s Research Institute. Oral informed consent was obtained from all focus group participants. | Yes, thematic analysis was conducted. The first author developed an initial coding scheme that was condensed and checked with participants in the obstetrician and patient focus groups. Furthermore, the second author independently coded the transcripts and compared the codes with the first author. | Yes, the researchers highlight that bereavement care training is needed to support parents emotionally during stillbirth. Additionally, it is necessary for providers to reduce the stigma and taboo around stillbirth to improve patients’ care experiences. | The findings echo other studies demonstrating the need for protocols and training around bereavement care. Because stillbirth is often an unexpected clinical outcome, knowledge of and comfortability handling stillbirth varies across providers. The study also shows obstetricians’ pursuit of answers for patients when stillbirth occurs, particularly when there is concern for legal action. The search for answers may distract providers from grasping the full depth of patients’ grief, therefore leading patients to feel unsupported. | The findings provide helpful commentary on the struggle of obstetricians to serve as “counselors” after cases of stillbirth. Concerns about blame and legal issues cloud providers’ ability to emotionally support patients, leading providers to defer to other staff for patients’ emotional needs. The findings also highlight obstetricians’ frustration when searching for answers regarding stillbirth and their inability to provide more information/answers to patients. |
| 1 | Lappeman, M. & Swartz, L. | 2019 | Yes, to explore doctors’ responses to stillbirth in a resource-limited, impoverished setting in South Africa and to investigate the impacts of exposure to high rates of patient death and trauma on doctors. | Yes, semi-structured interviews examined the experiences of doctors working in an overworked, stressed healthcare system, the emotional impacts of this setting, and the influence on care provided. | Yes, semi-structured interviews were conducted with 8 doctors (n=2 obstetricians, n=2 obstetric trainees, n=4 medical doctors) working in the labour ward of a large metropolitan hospital. Interviews were conducted based on the free association narrative interview methodology in which the interviewer seeks to evoke memories with emotional content through open-ended conversation. | Yes, purposive sampling was conducted to recruit eight medical doctors. Participants were required to have worked in the labour ward for at least 4 months and to have provided care for at least one stillbirth case. | Yes, interviewer were conducted by a trained clinical psychologist in the labour ward of the hospital, though the length of the interviews nor privacy measures are mentioned. | Yes, the interviewer worked as a clinical psychologist in the same hospital as the participants and acknowledged that the previous relationship with participants was key to the FANI method. | Yes, ethical approval was obtained from the Research Ethics Committee and from the Department of Health and participants, though an informed consent process is not specified. | Yes, data were analyzed based on the FANI method to elicit emotions, thoughts, and motivations of participant. This analysis operates under the assumption that participants are guarded and defensive to protect against their emotions and trauma. It is not specified whether more than one researcher contributed to analysis. | No, it is not clear what the primary outcomes are. However, the author specifies that one key outcome is to acknowledge the complex political and social burdens healthcare providers experience when working in a culturally-divided community. | This research captures an important community not captured in other studies. Not only do healthcare providers work in a low-resource, violent, impoverished setting, but also in a community that is culturally divided. This study seeks to understand how the burden of these cultural divides influences the care provided. | This study uniquely layers the influence of cultural strain and the impacts of a stressed healthcare system on providers.  The results of this study discern the underlying tension providers feel with members of the community to whom they provide care. Doctors demonstrate a tendency to detach themselves to avoid emotional involvement with community members. This detachment causes lack of effort to engage with patients beyond physical care provided. There are competing needs to recognize the burden of cultural divides and resource limitations on doctors while also acknowledging that doctors need to improve their emotional involvement with patients.  One limitation is that it appears that only one researcher contribute to analysis, leaving the results to that researcher’s subjective interpretation. |
| 1 | Lawrence, E., Parekh, B., Owusu-Antwi, R., Newman, N., Russell, C., Beyuo, T., Yeboah, M., Oppong, S.A., Moyer, C.A. | 2024 | Yes, to understand obstetric providers’ views on seeking mental health support following poor clinical outcomes, particularly maternal and neonatal mortality. | Yes, focus groups were utilized to investigate the impact of mental health stigma on obstetric providers’ views about seeking mental health care after poor clinical outcomes. | Yes, five semi-structured focus groups were conducted with 52 obstetric providers (n=20 OB-GYNs) from two tertiary care centers in Ghana. Focus groups were conducted using an open-ended guide based on prior studies in Ghana and the lived experiences of researchers in Ghana. | Yes, purposive sampling was used to recruit participants with meaningful experiences and to cultivate a sample with a diversity of views. Recruitment occurred via departmental WhatsApp groups and written announcements. | Yes, focus groups lasting approximately 2 hours were facilitated by an experienced maternal and child health researcher in Ghana. Focus groups with 8-14 participants were hosted in a private hotel conference room to protect participant confidentiality. | Not sure, no prior relationship is mentioned between participants and researchers. | Yes, ethical approval was obtained from institutional review boards at both tertiary care hospitals. Researchers obtained written informed consent from all participants and discussed the potential for psychological distress from reliving poor/traumatic outcomes. | Yes, though no specific method is mentioned, two researchers generated a codebook and developed a comprehensive list of codes that could be used for all transcripts. Researchers organized codes into basic themes and then overarching themes. | Yes, the researcher concluded that stigma around mental health support posed a significant barrier to obstetric providers seeking help. Though providers discussed the negative effects of adverse clinical outcomes, their overarching desire was to continue with work and move forward from incidents. | These findings provide crucial insight into providers’ experiences with poor clinical outcomes in lower-resource settings, since most research on perinatal loss occurs in high-income countries. Additionally, this study highlights the influence of cultural norms on the response of healthcare providers to trauma/adverse outcomes; since Ghana as a society promotes emotional restraint, obstetric providers are expected to contain their emotional reactions. | The larger sample size of OB-GYNs (n=20) provides a better picture of the obstetrician perspective. Systemic factors due to limited resources compounded frustration and feelings of impotence among providers, since many felt that the maternal and neonatal deaths they experienced could have been prevented with better resources (i.e., lower patient volumes and improved infrastructural support).  Like studies in high-resource settings, this study supports the need for increased peer support and spaces for sharing experiences, though seeking help is against Ghanaian cultural norms. Another novel theme was the perception amongst providers that it was acceptable for their patients to seek mental health support but not for them. |
| 1 | McNamara, K., Meaney, S., O’Connell, O., McCarthy, M., Greene, R.A., O’Donoghue, K. | 2017 | Yes, to explore the experiences of healthcare providers exposed to intrapartum death (IPD). | Yes, an open-ended questionnaire format investigated obstetricians’ experiences with IPD and gathered insights into the impact of IPD on care provision and possible support and education strategies for healthcare providers. | A questionnaire with both open- and closed-ended questions was administered to 89 healthcare professionals (n=11 obstetric consultants) in a tertiary-referral university-teaching hospital in Ireland. Questions were based on the previously published literature on stillbirth impact on healthcare providers. Of note, consultant obstetricians answered 9 additional questions regarding how they supported junior colleges during IPD. | Yes, all consultants were invited to participate, as well as NCHDs who worked on the labour ward between 2010 and 2015, though specific sampling method is not mentioned. | Yes, the primary author collected questionnaire responses electronically. All participants were given the opportunity to provide additional feedback with free text options in the survey. | Not significant, since the study was administered in questionnaire format. | Yes, ethical approval was obtained from the Clinical Research Ethics Committee, though an informed consent process is not specified. | Yes, open coding was conducted to draw out the underlying meanings of and similarities across the data. Initial codes were developed and then constructed into themes. | Yes, the findings clearly demonstrate that increased support and education is needed for healthcare providers following IPD. Negative personal impacts on healthcare providers include guilt, sadness, and isolation. | Yes, this study is unique in that it focuses on purely the obstetrician perspective as opposed to the perspective of nurses, midwives, and other health professionals. The focus on obstetricians draws out the major theme that obstetricians bear the burden of blame and guilt in IPD cases, which can lead to isolation and doubts in decision-making. Another important theme is the desire for peer support, which obstetricians desired in a non-judgmental environment. | The findings are limited by the questionnaire design; though the questions were open-ended, an interview format could elicit more thoughts/insights.  Despite this limitation, these findings highlight the unique personal impact/responsibility that doctors face, which can cause them to question their clinical skills. The burden of self-doubt poses implications for future clinical care. To reckon with IPD cases, obstetricians require increased time and peer/emotional support. |
| 1 | McNamara, K., Meaney, S., O’Donoghue, K. | 2018 | Yes, to understand obstetricians’ attitudes towards and responses to intrapartum fetal death. | Yes, semi-structured interviews discerned the experiences of obstetricians with intrapartum fetal death and how experiences impact care provided. | Yes, semi-structured interviews of 10 obstetricians in a tertiary university maternity ward were conducted based on a topic guide designed by the co-authors. Interviews also included discussions of different scenarios that helped generate feedback not elicited in the interviews. | Yes, purposive sampling was conducted to recruit obstetricians from a large tertiary maternity hospital in Ireland. Obstetricians were required to have experienced direct involvement with an intrapartum fetal death. Participants received an email invitation and five further reminders over 3 months. | Yes, interviews ranging from 33 to 101 minutes were conducted by the primary author and/or the co-author from November 2015 to December 2016. However, the location and privacy measures of the interviews are not specified. | Yes, it is acknowledged that the interviewer previously knew the participants since the interviewer was an obstetric trainee. | Yes, ethical approval was obtained from the Clinical Research Ethics Committee. Written consent was obtained from participants prior to interviews. Additionally, participants identities were only known by the researchers to ensure confidentiality. | Yes, interpretation phenomenological analysis was conducted by the primary author and consensus was reached with another author who independently analyzed the data. Themes were developed after consensus between the coders was reached. | Yes, the study clearly emphasize the negative emotional impacts and burden experienced by obstetricians involved in IPDs and highlights the need for further emotional support interventions. | This study echoes the results of other studies indicating the improved emotional support for obstetricians and increased recognition of the burden obstetricians experience when coping with adverse clinical outcomes like IPD.  Furthermore, this study reflects the sense of responsibility demonstrated across the literature. | Interestingly, this study draws out the theme that when dealing with IPD obstetricians feel the need to set their emotions and needs aside in order to provide high-quality care. This forced emotional detachment contributes to obstetricians’ experiences as second victims of IPDs.  Not only do obstetricians need increased support, but they also need institutional protection against the pervasive blame culture.  Like other studies, this study demonstrated that there were not significant differences between the experiences of consultant and trainees.  One limitation of this study is the previous relationship between the participant and interviewer, which may also have been influenced by the interviewer’s status as a trainee. |
| 1 | Montero, S.M.P., Sanchez, J.M.R., Montoro, C.H., Crespo, M.L., Jaen, A.G.V., Tirado, M.B.R | 2011 | Yes, to explore the experiences of healthcare professionals managing perinatal loss. | Yes, semi-structured interviews were conducted with 19 health professionals to understand their experiences of and the action strategies employed while managing cases of perinatal loss. | Yes, semi-structured interviews with 19 healthcare professionals (n=2 obstetricians) used an interview guide based on various theme areas, each introduced with an open question. | Not sure, a specific recruitment strategy is not provided; however, participants were recruited from the maternal-infant unit of a provincial hospital in Spain. | Yes, interviews lasting from 60 to 90 minutes were conducted in participants’ workplace, with the time and location selected by participants. Data collection occurred between April 2007 and September 2008. However, the training/qualifications of the interviewer(s) is not specified. | Not sure, it is not specified whether the interviewer and participants established rapport prior to interviews. | Yes, ethical approval was obtained from the Research Committee at the hospital and the Institutional Review Board. Written informed consent was obtained from all study participants. | Yes, phenomenological analysis was conducted using NVivo. The researchers identified meaning units and grouped them into common themes that were broken into sub-themes. | Yes, the findings indicate that providers’ lack of knowledge and skills to manage perinatal loss lead to negative experiences. | Yes, the findings support other studies demonstrating the need for parental bereavement training and guideline development. Additionally, this study draws out the important point that deficits in knowledge contribute to feelings of professional incompetence. The findings also echo themes of blame, as well as feelings of guilt and failure. | The small sample size of obstetricians (n=2) suggests that further research is needed to explore the physician point of view.  The findings contribute to the body of knowledge indicating that obstetricians lack the skills and resources to deal with the emotional aspect of patient care. Concentration on the physical side of patient care compromises obstetricians’ ability to empathize with patients. Additionally, findings suggest that physicians may avoid emotional care because they do not know what to say. Physicians experience impotence over the situation and feelings of incompetence that may prevent them from sharing in patients’ sorrow. |
| 1 | Nuzum, D., Meaney, S., O’Donoghue, K. | 2014 | Yes, to investigate the impact of stillbirth on obstetrician-gynecologists. | Yes, semi-structured interviews were conducted to examine how the personal effects of stillbirth influenced care provided by OB-GYNs. | Yes, semi-structured interviews were conducted with 8 OB-GYNs using a topic guide designed to discern personal and professional experiences with stillbirth. Following open questions provided by the topic guide, physicians were asked to describe how they would respond to a scene in which they confirmed stillbirth for a couple at 38 weeks gestation. | Yes, purposive sampling of OB-GYNs form a university maternity hospital in Ireland was utilized to develop a group with gender and specialization balance, as well as a balance of individuals working in academic and clinical settings. | Yes, interviews ranging from 27 to 58 minutes occurred in the participants’ workplace at times selected by participants. | Yes, it was acknowledged that one researcher who worked as a consultant OB-GYN and therefore was not aware of participants’ identities. | Yes, ethical approval was obtained from the Clinical Research Ethics Committee, though informed consent was not mentioned. | Yes, interpretation phenomenological analysis was conducted, a theory which seeks to examine participants’ experiences and how they make sense of them. Themes and subthemes were developed based on consensus among researchers. NVivo was used for analysis/data management. | Yes, the findings emphasize that OB-GYNs lack training in perinatal bereavement care. This deficit in training affects future care provision and influences personal effects on OB-GYNs. | This study echoes the finding that training in bereavement care is essential to support OB-GYNs experiencing perinatal loss, so that they may provide a high quality of care to patients.  Additionally, the data highlight the personal responsibility felt by OB-GYNs following stillbirth, which can cause feelings of doubt or that something was missed.  Another important contribution of this study is the emphasis on empathetic care; the draining nature of providing empathetic, human-centered care increases the importance of emotional support for providers. There was a shared sentiment that some OB-GYNs were “out of their depth” when providing emotional care. | An important theme in this study is the high, and often unrealistic, standards placed on OB-GYNs. This burden contributes both to the personal responsibility felt by OB-GYNs after stillbirth and to legal concerns. Legal concerns generate fear that can hinder care provision.  The feelings of sadness, anger, blame, isolation, and disappointment experienced by this sample of OB-GYNs is reflected in other settings, whether high- or low-resource. Negative emotional impacts may detract from providers’ ability to care for patients emotionally.  The advantage of this study is that it focuses solely on the experiences of OB-GYNs. However, a limitation is that interpretative phenomenological analysis focuses on individual experiences as opposed to populations, so these results cannot be generalized to the broader OB-GYN popuation. |
| 1+2 | Petrites, A., Mullan, P., Spangenberg, K., Gold, K. | 2016 | Yes, to understand how providers in low-resource settings cope with high rates of perinatal loss. | Yes, semi-structured interviews were conducted to investigate how physicians and midwives make sense of perinatal death and to identify coping strategies. | Yes, semi-structuerd interviews with 36 healthcare providers (n=13 OB-GYNs) from a large tertiary-referral teaching hospital in Ghana discerned emotional effects of perinatal death and identified coping strategies, though it is not specified how the interview guide was developed. | Yes, participants recruited from staff registries based and invited to participant; subsequent participants were identified via snowball sampling. The researchers recruited participants to ensure a diversity of views, specialties, gender, level of training/experiences, etc. | Yes, interviews lasting about 30 minutes were conducted by 8 medical students trained in qualitative interviewing techniques; private rooms in the hospital or medical school were used to ensure confidentiality. | Yes, it is specified that interviewers had limited contact with participants. | Yes, ethical approval was received from the Institutional Review Board and the Committee on Human Research Publication and Ethics. Verbal informed consent was obtained from all participants. Notably, participants were offered a $3 incentive. | Yes, though a specific coding method is not mentioned, two researchers identified themes after reading the transcripts; they further categorized the themes into discrete themes after multiple iterations. | Yes, the overarching theme was that healthcare providers desired to move forward through trauma. However, a need for perinatal loss and coping skill training still exists. | With the unique perspective of the low-resource setting, this study draws out the unique theme that providers desire to continue pushing forward after perinatal loss. After loss, providers were galvanized to provide the best care possible to the next patient. In contrast to other studies and settings, these findings show that some providers believed there was nothing they could do in perinatal loss situations, and that they had performed to the best of their ability. | Uniquely, this study categorizes providers into low- and high-self efficacy, with low self-efficacy providers expressing they had minimal control over perinatal death and high-efficacy providers felt motivated to take action in the wake of loss.  One interesting theme was the influence of systemic factors, i.e., issues with facilities, equipment, and staffing, that hindered healthcare providers’ ability to provide high quality of care while also preventing them from taking time to process or grieve.  Similar to in other settings, some providers demonstrated emotional avoidance and detachment from perinatal loss. In the low-resource setting in particular, emotional detachment might reflect providers’ desire to move on to the next case and provide the best care possible to future patients/avoid future losses. |
| 1 | Power, S., Meaney, S., O’Donoghue, K. | 2020 | Yes, to explore fetal medicine specialists’ (FMSs) experiences and management of pregnancies complicated by fatal fetal anomaly (FFA). | Yes, semi-structured interviews were conducted to understand experiences and care management of FFA, with a focus on providing termination of pregnancy. | Yes, semi-structured interviews of 10 fetal-medicine specialists from five fetal medicine units in Ireland used questions to investigate how FMSs made sense of their lived experiences, specifically in light of the implementation of termination of pregnancy. The authors developed the interview guide with contributions from the Pregnancy Loss Research Group. Additionally, a topic guide helped facilitate interviews. | Yes, the researchers utilized purposive sampling to obtain participants who were currently practicing as FMSs. Potential participants were provided with leaflets. | Yes, interviews ranging from 36 to 71 minutes were conducted from November 2019 to January 2020 by one researcher, though the location of interviews is not specified. | Yes, it is specified that there was no relationship between the interviewer and participants. | Yes, ethical approval was obtained from the Clinical Research Ethics Committee, though informed consent is not mentioned. | Yes, thematic analysis was conducted by one researcher who discussed with a second researcher to achieve consensus. The initial codes were categorized into themes and then subthemes. NVivo was used for data analysis. | Yes, though the results and conclusion are cumbersome, they are divided into clear themes that demonstrate the anxiety and fear felt by FMSs amidst FFA diagnosis and care provision for termination of pregnancy. | Like the existing literature, this study emphasizes the need for peer support, especially when providing care for FFA.  In contrast to other studies, this study examines a setting in which legal concerns are heightened, since the interviews took place following the implementation of new laws allowing FMSs to provide termination of pregnancy. FMSs experienced increased pressure to provide a correct diagnosis, and many expressed fear that the diagnosis was “not fatal enough” to provide termination of pregnancy care and that they would suffer legal consequences. | In light of Ireland’s unique legal climate, FMSs showed high anxiety related to giving permission for termination of pregnancy. The weight of responsibility was more burdensome in the context of increased legal and media ramifications. Additionally, FMSs discussed the lack of institutional support and preparedness to deal with the implementation of new legislation.  Unique to the Irish setting, FMSs felt ostracized and unsupported by conscientious objectors to termination of pregnancy, even amongst coworkers. |
| 1+2 | Serafim, T.C., Camilo, B.H.N., Carizani, M.R., Gervasio, M., Carlos, D.M., Salim, N.R. | 2021 | Yes, to understand how health professionals manage intrauterine fetal death for patients, with a focus on coping mechanisms and strategies for care provision. | Yes, semi-open interviews were conducted to understand health professionals’ experiences with intrauterine fetal death, given the lack of guidelines for care management and lack of spaces to share with peers. | Yes, semi-open interviews based on guiding questions were conducted with 11 professionals (n=3 obstetricians) from the countryside of Sao Paolo, Brazil, though it is not clear how the guiding questions were developed. | Yes, a snowball sampling method was used, with the first professional being contacted during shift change. Professionals were required to have at least one year of experience in obstetric care. | Yes, interviews lasting from 20 to 60 minutes were administered by a trained undergarduate nursing student with supervision from an advisor. Participants selected the location of interviews. Data collection occurred from August 2018 to February 2019. | Yes, it is specified that no rapport was established between the interviewer and participants prior to interviews. | Yes, ethical approval was obtained from the Research Ethics Committee. Written consent was obtained from all participants. | Yes, thematic content analysis was conducted, though it is not clear which or how many researchers participated in analysis. Additionally, it is not specified how final themes were developed. | Yes, the researcher clearly show that strategies and guidelines for providing care and coping with intrauterine fetal death are needed. Additionally, feelings of impotence/helplessness were apparent as providers struggled to care for patients’ emotional needs | This study contribute to the body of work demonstrating the need for bereavement care training and support for providers. Furthermore, these findings reflect the tendency of providers to detach emotionally and avoid managing patients from an empathetic perspective to avoid their own pain and suffering. | Due to lack of training and support, providers do not know what to say or how to act in situations of intrauterine fetal death and resort to avoiding the emotional aspect of care. Because of feelings of impotence, providers often focus on investigating cases and searching for answers as opposed to supporting patients emotionally.  Another important theme is that physicians serve as the first line of communication with patients and experience the burden of giving news. While communication between patients and providers is crucial in situations involving IFD, physicians often fail to treat patients with empathy because they are overburdened and avoid their own pain/suffering to move forward with their jobs. |
| 1 | Sheen, K., Goodfellow, L., Balling, K., Rymer, J., Weeks, A., Spiby, H., Slade, P. | 2022 | Yes, to identify which obstetric events are most traumatic to obstetricians and to understand the factors that contribute to the degree of trauma experienced among members of the Royal College of Obstetricians and Gynaecologists. | Yes, semi-structured interviews were conducted to understand their experiences with traumatic clinical outcomes and to discern what made events traumatic. | Yes, semi-structured interviews with 43 obstetric consultants, trainees, and RCOG members using an interview guide, though it is not specified how the interview guide was developed. | Yes, purposive sampling was used to recruit RCOG members from a larger questionnaire study of traumatic obstetric experiences. Survey participants who indicated willingness to partake in interviews were contacted. Interview participants were divided into high and low post-traumatic stress disorder groups to gauge the different factors influencing traumatic outcomes for each group. | Yes, interviews lasting about 45 minutes were conducted via telephone by a clinical psychologist. Participants selected the time of interviews and were aware of the interviewer’s capacity as a clinical psychologist and research associate. | Not sure, it is not specified whether the interviewer established rapport with the participants beyond contacting them with study information prior to the interview. | Yes, ethical approval was obtained from the Ethics Committee. Informed consent was obtained from participants prior to interviews. | Yes, template analysis was conducted on the high PTSD and low PTSD groups separately. The template used for analysis concentrate on what made events traumatic, the impacts on healthcare providers, how providers managed the impacts, what prevented management of traumatic impacts, and what healthcare providers desired in the future. It is not mentioned whether more than one researcher conducted analysis. | Yes, the study clearly identifies that maternal and neonatal death/stillbirth, haemorrhage, and difficult deliveries were the most traumatic events. Additionally, the study demonstrates that the factors contributing to trauma were unpredictability of events, perceived preventability, and high emotionality. | Importantly, this study suggests that stillbirth is one of the most traumatic events experienced by obstetricians, demonstrating that further research on and support for doctors experiencing stillbirth is validated. Additionally, this research focuses on the specific factors that make stillbirth and other clinical outcomes traumatic, not just on the experiences of individual physicians. | This study reinforces the trauma obstetricians face when managing stillbirth cases. Additionally, the results call for increased training to manage traumatic events and for development of support strategies for providers.  Like other papers, this study highlights the immense sense of responsibility obstetricians feel in traumatic situations and how this burden impacts the care provided. This study also identifies feelings of professional helplessness as an outcome of traumatic clinical encounters.  One limitation of this study is that it is not focused on stillbirth specifically, since all potentially traumatic events were investigated. |

Legend: 1= Does the study describe experiences of obstetricians with stillbirth management?, 2= Does the study explain how managing stillbirth affects obstetrician experiences?, 3= Does the study explain how the effects of stillbirth management impact obstetricians across settings?
