# Supplementary material for: How obstetricians experience stillbirth and perinatal loss: a systematic review and meta-synthesis
Source: AJOG Glob Rep. 2025 Feb 16;5(2):100465. doi: 10.1016/j.xagr.2025.100465 (PMC11931382; doi:10.1016/j.xagr.2025.100465)
Supplement: Supplementary file 2 [file mmc2.docx]

Supplementary Appendix A: Search Strategy

How do obstetricians experience stillbirth and perinatal loss?

| Medline via Ovid | 2906 |
| --- | --- |
| Embase via Ovid | 3278 |
| CINAHL via Ebsco | 3642 |
| PsycINFO via Ebsco | 454 |
| Scopus | 5461 |
| Web of Science Core Collection | 2112 |
| ASSIA via Proquest | 119 |
| Total | 18052 |
| Total dedupliated |  |

Searches run 7 April 2024

No date or language limits.

# Medline

Ovid MEDLINE(R) and Epub Ahead of Print, In-Process, In-Data-Review & Other Non-Indexed Citations, Daily and Versions <1946 to November 29, 2023>

1 exp patient satisfaction/ 100095

2 (attitude* or view* or perception* or satisf* or experienc* or perceiv* or influenc* or facilitat* or barrier*).ti,ab,kw,kf. 5212671

3 (qualitative or narrative or semi-structured or unstructured or informal or structured or interview* or questionnaire* or survey* or focus group* or ethnograph* or field work or naturalistic or phenomenology).ti,ab,kw,kf. 2113425

4 exp Interview/ or exp Qualitative Research/ or exp Focus Groups/ or exp Anthropology, Cultural/ or exp "Surveys and Questionnaires"/ 1498837

5 ((obstetric adj (care* or clinic*)) or antenatal or antepartum or high-risk pregnancy clinic* or (outpatient adj (care or clinic*)) or (prenatal adj (care or clinic*)) or (public health* centre* or public health* center*) or midwif* or midwive*).ti,ab,kw,kf. 143423

6 exp "Obstetrics and Gynecology Department, Hospital"/ or Delivery, Obstetric/ or exp Obstetricians/ or exp Prenatal Care/ or exp Perinatal Care/ or Ambulatory Care/ or exp Midwifery/ 137618

7 ((high risk* adj3 (pregnan* or gestation or birth or childbirth)) or ((gestation* or pregnan*) adj2 diabet*) or f?etal macrosomia* or ((f?etal or f?etus or f?etous or embryo* or perinat* or neonat*) adj2 (death* or mortality)) or stillborn or (still adj (born or birth*)) or ((birth or f?etal*) adj2 (defect* or abnormal*)) or (f?etal adj alcohol) or FASD or FASDS or ((f?etal or f?etus or intrauterine) adj (growth restrict* or restrict* growth*)) or ((f?etal or f?etus) adj (hypoxi* or anoxi*)) or ((pregnan* or gestation*) adj2 hypertensi*) or pre-eclampsia or eclampsia or HELLP syndrome or hyperemesis gravidarum or pregnancy pernicious vomiting or abrupti* placenta* or premature rupture* f?etal membrane* or preterm prom or (premature* adj (labo?r or birth* or childbirth* or contraction*)) or ((increta or accreta or precreta or previa) adj placenta*) or oligohydramnios or ((pregnan* or maternal) adj (phenylketonuria or phenylalanine or pku)) or (placenta* adj (insufficien* or fail*)) or (pregnan* adj2 complicat*) or adverse birth outcome* or ((cystic fibrosis or lupus or thyroid disease* or thyroid disorder* or hyperthyroid* or hypothyroid*) adj3 (pregnan* or childbirth* or labo?r*)) or (multiple adj (pregnan* or gestation*)) or multi f?etal gestation* or twin* or triplet*).ti,ab,kw,kf. 287360

8 exp pregnancy, high-risk/ or exp diabetes, gestational/ or exp fetal death/ or exp fetal alcohol spectrum disorders/ or exp fetal growth retardation/ or exp fetal hypoxia/ or exp hypertension, pregnancy-induced/ or exp hyperemesis gravidarum/ or exp abruptio placentae/ or exp fetal membranes, premature rupture/ or exp obstetric labor, premature/ or exp placenta accreta/ or exp placenta previa/ or exp oligohydramnios/ or exp perinatal death/ or exp phenylketonuria, maternal/ or exp placental insufficiency/ or exp pregnancy complications, cardiovascular/ or exp pregnancy complications, hematologic/ or exp pregnancy in diabetes/ or exp pregnancy, multiple/ 197832

9 (1 or 2) and (3 or 4) and (5 or 6) and (7 or 8) 2906

# Embase

Embase <1974 to 2023 November 29>

1 exp *patient satisfaction/ 29296

2 (attitude* or view* or perception* or satisf* or experienc* or perceiv* or influenc* or facilitat* or barrier*).ti,ab. 6545378

3 (qualitative or narrative or semi-structured or unstructured or informal or structured or interview* or questionnaire* or survey* or focus group* or ethnograph* or field work or naturalistic or phenomenology).ti,ab. 2721107

4 exp *Interview/ or exp *Qualitative Research/ or exp *qualitative analysis/ or exp *ethnography/ or exp *cultural anthropology/ or exp *health care survey/ or exp *questionnaire/ 112618

5 ((obstetric adj (care* or clinic*)) or antenatal or antepartum or high-risk pregnancy clinic* or (outpatient adj (care or clinic*)) or (prenatal adj (care or clinic*)) or (public health* centre* or public health* center*) or midwif* or midwive*).ti,ab. 198741

6 exp *obstetrics/ or exp *obstetric delivery/ or exp *Obstetrician/ or exp *Prenatal Care/ or exp *Perinatal Care/ or exp *outpatient department/ or exp *Midwife/ 220962

7 ((high risk* adj3 (pregnan* or gestation or birth or childbirth)) or ((gestation* or pregnan*) adj2 diabet*) or f?etal macrosomia* or ((f?etal or f?etus or f?etous or embryo* or perinat* or neonat*) adj2 (death* or mortality)) or stillborn or (still adj (born or birth*)) or ((birth or f?etal*) adj2 (defect* or abnormal*)) or (f?etal adj alcohol) or FASD or FASDS or ((f?etal or f?etus or intrauterine) adj (growth restrict* or restrict* growth*)) or ((f?etal or f?etus) adj (hypoxi* or anoxi*)) or ((pregnan* or gestation*) adj2 hypertensi*) or pre-eclampsia or eclampsia or HELLP syndrome or hyperemesis gravidarum or pregnancy pernicious vomiting or abrupti* placenta* or premature rupture* f?etal membrane* or preterm prom or (premature* adj (labo?r or birth* or childbirth* or contraction*)) or ((increta or accreta or precreta or previa) adj placenta*) or oligohydramnios or ((pregnan* or maternal) adj (phenylketonuria or phenylalanine or pku)) or (placenta* adj (insufficien* or fail*)) or (pregnan* adj2 complicat*) or adverse birth outcome* or ((cystic fibrosis or lupus or thyroid disease* or thyroid disorder* or hyperthyroid* or hypothyroid*) adj3 (pregnan* or childbirth* or labo?r*)) or (multiple adj (pregnan* or gestation*)) or multi f?etal gestation* or twin* or triplet*).ti,ab. 345167

8 exp *high risk pregnancy/ or exp *pregnancy diabetes mellitus/ or exp *fetus death/ or exp *fetal alcohol syndrome/ or exp *intrauterine growth retardation/ or exp *fetus hypoxia/ or exp *maternal hypertension/ or exp *hyperemesis gravidarum/ or exp *solutio placentae/ or exp *premature fetus membrane rupture/ or exp *premature labor/ or exp *placenta accreta/ or exp *placenta previa/ or exp *oligohydramnios/ or exp *perinatal death/ or exp *perinatal mortality/ or exp *phenylketonuria/ or exp *placenta insufficiency/ or exp *pregnancy complications/ or exp *multiple pregnancy/ 171127

9 (1 or 2) and (3 or 4) and (5 or 6) and (7 or 8) 3278

# Scopus

TITLE-ABS-KEY ( attitude* OR view* OR perception* OR satisf* OR experienc* OR perceiv* OR influenc* OR facilitat* OR barrier* ) AND TITLE-ABS-KEY ( qualitative OR narrative OR semi-structured OR unstructured OR informal OR structured OR interview* OR questionnaire* OR survey* OR "focus group*" OR ethnograph* OR "field work" OR naturalistic OR phenomenology ) AND TITLE-ABS-KEY ( ( obstetric W/1 ( care* OR clinic* ) ) OR antenatal OR antepartum OR "high-risk pregnancy clinic*" OR ( outpatient W/1 ( care OR clinic* ) ) OR ( prenatal W/1 ( care OR clinic* ) ) OR ( "public health* centre*" OR "public health* center*" ) OR midwif* OR midwive* ) AND TITLE-ABS-KEY ( ( "high risk*" W/3 ( pregnan* OR gestation OR birth OR childbirth ) ) OR ( ( gestation* OR pregnan* ) W/2 diabet* ) OR "f?etal macrosomia*" OR ( ( f?etal OR f?etus OR f?etous OR embryo* OR perinat* OR neonat* ) W/2 ( death* OR mortality ) ) OR stillborn OR ( still W/1 ( born OR birth* ) ) OR ( ( birth OR f?etal* ) W/2 ( defect* OR abnormal* ) ) OR ( "f?etal alcohol" ) OR fasd OR fasds OR ( ( f?etal OR f?etus OR intrauterine ) W/1 ( "growth restrict*" OR "restrict* growth*" ) ) OR ( ( f?etal OR f?etus ) W/1 ( hypoxi* OR anoxi* ) ) OR ( ( pregnan* OR gestation* ) W/2 ( hypertensi* ) ) OR pre-eclampsia OR eclampsia OR "HELLP syndrome" OR "hyperemesis gravidarum" OR "pregnancy pernicious vomiting" OR "abrupti* placenta*" OR "premature rupture* f?etal membrane*" OR "preterm prom" OR ( premature* W/1 ( labo?r OR birth* OR childbirth* OR contraction* ) ) OR ( ( increta OR accreta OR precreta OR previa ) W/1 placenta* ) OR oligohydramnios OR ( ( pregnan* OR maternal ) W/1 ( phenylketonuria OR phenylalanine OR pku ) ) OR ( placenta* W/1 ( insufficien* OR fail* ) ) OR ( pregnan* W/2 complicat* ) OR "adverse birth outcome*" OR ( ( "cystic fibrosis" OR lupus OR "thyroid disease*" OR "thyroid disorder*" OR hyperthyroid* OR hypothyroid* ) W/3 ( pregnan* OR childbirth* OR labo?r* ) ) OR ( multiple W/1 ( pregnan* OR gestation* ) ) OR "multi f?etal gestation*" OR twin* OR triplet* )

# Web of Science Core Collection

# Web of Science Search Strategy (v0.1)

# Database: Web of Science Core Collection

# Entitlements:

- WOS.IC: 1993 to 2023

- WOS.CCR: 1985 to 2023

- WOS.SCI: 1900 to 2023

- WOS.AHCI: 1975 to 2023

- WOS.BHCI: 2008 to 2023

- WOS.BSCI: 2008 to 2023

- WOS.ESCI: 2018 to 2023

- WOS.ISTP: 1990 to 2023

- WOS.SSCI: 1956 to 2023

- WOS.ISSHP: 1990 to 2023

# Searches:

1: TS=(qualitative or narrative or semi-structured or unstructured or informal or structured or interview* or questionnaire* or survey* or “focus group*” or ethnograph* or field work or naturalistic or phenomenology) Date Run: Thu Nov 30 2023 12:15:30 GMT+0000 (Greenwich Mean Time) Results: 10813547

2: TS= ((obstetric near/1 (care* or clinic*)) or antenatal or antepartum or “high-risk pregnancy clinic*” or (outpatient near/1 (care or clinic*)) or (prenatal near/1 (care or clinic*)) or (“public health* centre*” or “public health* center*”) or midwif* or midwive*) Date Run: Thu Nov 30 2023 12:15:39 GMT+0000 (Greenwich Mean Time) Results: 141904

3: TS=(attitude* or view* or perception* or satisf* or experienc* or perceiv* or influenc* or facilitat* or barrier*) Date Run: Thu Nov 30 2023 12:15:45 GMT+0000 (Greenwich Mean Time) Results: 10974819

4: TS= ( ("high risk*" near/3 (pregnan* or gestation or birth or childbirth)) or ((gestation* or pregnan*) near/2 diabet*) or “f$etal macrosomia*” or ((f$etal or f$etus or f$etous or embryo* or perinat* or neonat*) near/2 (death* or mortality)) or stillborn or (still near/1 (born or birth*)) or ((birth or f$etal*) near/2 (defect* or abnormal*)) or ("f$etal alcohol") or FASD or FASDS or ((f$etal or f$etus or intrauterine) near/1 ("growth restrict*" or "restrict* growth*")) or ((f$etal or f$etus) near/1 (hypoxi* or anoxi*)) or ((pregnan* or gestation*) near/2 (hypertensi*)) or pre-eclampsia or eclampsia or "HELLP syndrome" or "hyperemesis gravidarum" or "pregnancy pernicious vomiting" or "abrupti* placenta*" or "premature rupture* f$etal membrane*" or "preterm prom" or (premature* near/1 (labo$r or birth* or childbirth* or contraction*)) or ((increta or accreta or precreta or previa) near/1 placenta*) or oligohydramnios or ((pregnan* or maternal) near/1 (phenylketonuria or phenylalanine or pku)) or (placenta* near/1 (insufficien* or fail*)) or (pregnan* near/2 complicat*) or "adverse birth outcome*" or (("cystic fibrosis" or lupus or "thyroid disease*" or "thyroid disorder*" or hyperthyroid* or hypothyroid*) near/3 (pregnan* or childbirth* or labo$r*)) or (multiple near/1 (pregnan* or gestation*)) or "multi f$etal gestation*" or twin* or triplet*) Date Run: Thu Nov 30 2023 12:15:53 GMT+0000 (Greenwich Mean Time) Results: 464014

5: #4 AND #3 AND #2 AND #1 Date Run: Thu Nov 30 2023 12:15:59 GMT+0000 (Greenwich Mean Time) Results: 2112

# ASSIA

| Set# | Searched for | Databases | Results |
| --- | --- | --- | --- |
| S1 | title((attitude* or view* or perception* or satisf* or experienc* or perceiv* or influenc* or facilitat* or barrier*)) OR abstract((attitude* or view* or perception* or satisf* or experienc* or perceiv* or influenc* or facilitat* or barrier*)) | Applied Social Sciences Index & Abstracts (ASSIA) | 482306 |
| S2 | title((qualitative or narrative or semi-structured or unstructured or informal or structured or interview* or questionnaire* or survey* or ("focus group" OR "focus groups") or ethnograph* or “field work” or naturalistic or phenomenology) ) OR abstract((qualitative or narrative or semi-structured or unstructured or informal or structured or interview* or questionnaire* or survey* or ("focus group" OR "focus groups") or ethnograph* or “field work” or naturalistic or phenomenology) ) | Applied Social Sciences Index & Abstracts (ASSIA) | 343132 |
| S3 | title(((obstetric near/1 (care* or clinic*)) or antenatal or antepartum or “high-risk pregnancy clinic*” or (outpatient near/1 (care or clinic*)) or (prenatal near/1 (care or clinic*)) or (“public health* centre*” or “public health* center*”) or midwif* or midwive*) ) OR abstract(((obstetric near/1 (care* or clinic*)) or antenatal or antepartum or “high-risk pregnancy clinic*” or (outpatient near/1 (care or clinic*)) or (prenatal near/1 (care or clinic*)) or (“public health* centre*” or “public health* center*”) or midwif* or midwive*) ) | Applied Social Sciences Index & Abstracts (ASSIA) | 17256 |
| S4 | title((("high risk" OR "high risks") near/3 (pregnan* or gestation or birth or childbirth)) or ((gestation* or pregnan*) near/2 diabet*) or “fetal macrosomia*” or “foetal macrosomia*” ) OR abstract((("high risk" OR "high risks") near/3 (pregnan* or gestation or birth or childbirth)) or ((gestation* or pregnan*) near/2 diabet*) or “fetal macrosomia*” or “foetal macrosomia*” ) | Applied Social Sciences Index & Abstracts (ASSIA) | 791 |
| S5 | title(((fetal* or foetal* or fetus* or foetus* or fetous* or foetous* or embryo* or perinat* or neonat*) near/2 (death* or mortality)) or stillborn or (still near/1 (born or birth*)) or ((birth or fetal* or foetal*) near/2 (defect* or abnormal*))) OR abstract(((fetal* or foetal* or fetus* or foetus* or fetous* or foetous* or embryo* or perinat* or neonat*) near/2 (death* or mortality)) or stillborn or (still near/1 (born or birth*)) or ((birth or fetal* or foetal*) near/2 (defect* or abnormal*))) | Applied Social Sciences Index & Abstracts (ASSIA) | 1845 |
| S6 | title(("fetal alcohol" or "foetal alcohol") or FASD or FASDS or ((fetal* or foetal* or fetus* or foetus* or fetous* or foetous* or intrauterine) near/1 (("growth restriction") or "restrict* growth*")) or ((fetal* or foetal* or fetus* or foetus* or fetous* or foetous*) near/1 (hypoxi* or anoxi*))) OR abstract(("fetal alcohol" or "foetal alcohol") or FASD or FASDS or ((fetal* or foetal* or fetus* or foetus* or fetous* or foetous* or intrauterine) near/1 (("growth restriction") or "restrict* growth*")) or ((fetal* or foetal* or fetus* or foetus* or fetous* or foetous*) near/1 (hypoxi* or anoxi*))) | Applied Social Sciences Index & Abstracts (ASSIA) | 554 |
| S7 | title(((pregnan* or gestation*) near/2 (hypertensi*)) or pre-eclampsia or eclampsia or "HELLP syndrome" or "hyperemesis gravidarum" or "pregnancy pernicious vomiting" or "abrupti* placenta*" or "premature rupture* fetal membrane*" or "premature rupture* foetal membrane*" or "preterm prom" ) OR abstract(((pregnan* or gestation*) near/2 (hypertensi*)) or pre-eclampsia or eclampsia or "HELLP syndrome" or "hyperemesis gravidarum" or "pregnancy pernicious vomiting" or "abrupti* placenta*" or "premature rupture* fetal membrane*" or "premature rupture* foetal membrane*" or "preterm prom" ) | Applied Social Sciences Index & Abstracts (ASSIA) | 420 |
| S8 | title((premature* near/1 (labor or labour or birth* or childbirth* or contraction*)) or ((increta or accreta or precreta or previa) near/1 placenta*) or oligohydramnios or ((pregnan* or maternal) near/1 (phenylketonuria or phenylalanine or pku)) ) OR abstract((premature* near/1 (labor or labour or birth* or childbirth* or contraction*)) or ((increta or accreta or precreta or previa) near/1 placenta*) or oligohydramnios or ((pregnan* or maternal) near/1 (phenylketonuria or phenylalanine or pku)) ) | Applied Social Sciences Index & Abstracts (ASSIA) | 293 |
| S9 | title((placenta* near/1 (insufficien* or fail*)) or (pregnan* near/2 complicat*) or "adverse birth outcome*") OR abstract((placenta* near/1 (insufficien* or fail*)) or (pregnan* near/2 complicat*) or "adverse birth outcome*") | Applied Social Sciences Index & Abstracts (ASSIA) | 706 |
| S10 | title((("cystic fibrosis" or lupus or ("thyroid disease" OR "thyroid diseases") or ("thyroid disorders") or hyperthyroid* or hypothyroid*) near/3 (pregnan* or childbirth* or labor* or labour*)) or (multiple near/1 (pregnan* or gestation*)) or "multi fetal gestation*" or "multi foetal gestation*" or twin* or triplet*) OR abstract((("cystic fibrosis" or lupus or ("thyroid disease" OR "thyroid diseases") or ("thyroid disorders") or hyperthyroid* or hypothyroid*) near/3 (pregnan* or childbirth* or labor* or labour*)) or (multiple near/1 (pregnan* or gestation*)) or "multi fetal gestation*" or "multi foetal gestation*" or twin* or triplet*) | Applied Social Sciences Index & Abstracts (ASSIA) | 5927 |
| S11 | [S4] OR [S5] OR [S6] OR [S7] OR [S8] OR [S9] OR [S10] | Applied Social Sciences Index & Abstracts (ASSIA)  These databases are searched for part of your query. | 10000 |
| S12 | [S1] AND [S2] AND [S3] AND [S11] | Applied Social Sciences Index & Abstracts (ASSIA)  These databases are searched for part of your query. | 199 |

# CINAHL

| 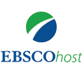 | Thursday, November 30, 2023 11:37:53 AM |
| --- | --- |

| **#** | **Query** | **Limiters/Expanders** | **Last Run Via** | **Results** |
| --- | --- | --- | --- | --- |
| S13 | S9 AND S10 AND S11 AND S12 | Expanders - Apply equivalent subjects  Search modes - Boolean/Phrase | Interface - EBSCOhost Research Databases  Search Screen - Advanced Search  Database - CINAHL | 3,642 |
| S12 | S7 OR S8 | Expanders - Apply equivalent subjects  Search modes - Boolean/Phrase | Interface - EBSCOhost Research Databases  Search Screen - Advanced Search  Database - CINAHL | 153,465 |
| S11 | S5 OR S6 | Expanders - Apply equivalent subjects  Search modes - Boolean/Phrase | Interface - EBSCOhost Research Databases  Search Screen - Advanced Search  Database - CINAHL | 153,890 |
| S10 | S3 OR S4 | Expanders - Apply equivalent subjects  Search modes - Boolean/Phrase | Interface - EBSCOhost Research Databases  Search Screen - Advanced Search  Database - CINAHL | 1,335,471 |
| S9 | S1 OR S2 | Expanders - Apply equivalent subjects  Search modes - Boolean/Phrase | Interface - EBSCOhost Research Databases  Search Screen - Advanced Search  Database - CINAHL | 1,405,931 |
| S8 | TI ((“high risk*” n3 (pregnan* or gestation or birth or childbirth)) or ((gestation* or pregnan*) n2 diabet*) or “f?etal macrosomia*” or ((f?etal or f?etus or f?etous or embryo* or perinat* or neonat*) n2 (death* or mortality)) or stillborn or (“still born*” or “still birth*”) or ((birth or f?etal*) n2 (defect* or abnormal*)) or (“f?etal alcohol”) or FASD or FASDS or ((f?etal or f?etus or intrauterine) n1 (growth restrict* or restrict* growth*)) or ((f?etal or f?etus) n1 (hypoxi* or anoxi*)) or ((pregnan* or gestation*) n2 hypertensi*) or pre-eclampsia or eclampsia or “HELLP syndrome” or “hyperemesis gravidarum” or “pregnancy pernicious vomiting” or “abrupti* placenta*” or “premature rupture* f?etal membrane*” or “preterm prom” or (premature* n1 (labo?r or birth* or childbirth* or contraction*)) or ((increta or accreta or precreta or previa) n1 placenta*) or oligohydramnios or ((pregnan* or maternal) n1 (phenylketonuria or phenylalanine or pku)) or (placenta* n1 (insufficien* or fail*)) or (pregnan* n2 complicat*) or “adverse birth outcome*” or ((“cystic fibrosis” or lupus or “thyroid disease*” or “thyroid disorder*” or hyperthyroid* or hypothyroid*) n3 (pregnan* or childbirth* or labo?r*)) or (multiple n1 (pregnan* or gestation*)) or “multi f?etal gestation*” or twin* or triplet*) or AB ((“high risk*” n3 (pregnan* or gestation or birth or childbirth)) or ((gestation* or pregnan*) n2 diabet*) or “f?etal macrosomia*” or ((f?etal or f?etus or f?etous or embryo* or perinat* or neonat*) n2 (death* or mortality)) or stillborn or (“still born*” or “still birth*”) or ((birth or f?etal*) n2 (defect* or abnormal*)) or (“f?etal alcohol”) or FASD or FASDS or ((f?etal or f?etus or intrauterine) n1 (growth restrict* or restrict* growth*)) or ((f?etal or f?etus) n1 (hypoxi* or anoxi*)) or ((pregnan* or gestation*) n2 hypertensi*) or pre-eclampsia or eclampsia or “HELLP syndrome” or “hyperemesis gravidarum” or “pregnancy pernicious vomiting” or “abrupti* placenta*” or “premature rupture* f?etal membrane*” or “preterm prom” or (premature* n1 (labo?r or birth* or childbirth* or contraction*)) or ((increta or accreta or precreta or previa) n1 placenta*) or oligohydramnios or ((pregnan* or maternal) n1 (phenylketonuria or phenylalanine or pku)) or (placenta* n1 (insufficien* or fail*)) or (pregnan* n2 complicat*) or “adverse birth outcome*” or ((“cystic fibrosis” or lupus or “thyroid disease*” or “thyroid disorder*” or hyperthyroid* or hypothyroid*) n3 (pregnan* or childbirth* or labo?r*)) or (multiple n1 (pregnan* or gestation*)) or “multi f?etal gestation*” or twin* or triplet*) | Expanders - Apply equivalent subjects  Search modes - Boolean/Phrase | Interface - EBSCOhost Research Databases  Search Screen - Advanced Search  Database - CINAHL | 70,938 |
| S7 | (MH "Pregnancy, High Risk") OR (MH "Diabetes Mellitus, Gestational") OR (MH "Perinatal Death") OR (MH "Fetal Alcohol Syndrome") OR (MH "Fetal Growth Retardation") OR (MH "Fetal Anoxia") OR (MH "Hypoxia-Ischemia, Brain, Neonatal") OR (MH "Pregnancy-Induced Hypertension+") OR (MH "Hyperemesis Gravidarum") OR (MH "Abruptio Placentae") OR (MH "Placenta Accreta") OR (MH "Fetal Membranes, Premature Rupture+") OR (MH "Labor, Premature") OR (MH "Placenta Praevia") OR (MH "Oligohydramnios") OR (MH "Phenylketonuria, Maternal") OR (MH "Placental Insufficiency") OR (MH "Pregnancy Complications+") OR (MH "Pregnancy in Diabetes+") OR (MH "Pregnancy, Multiple+") | Expanders - Apply equivalent subjects  Search modes - Boolean/Phrase | Interface - EBSCOhost Research Databases  Search Screen - Advanced Search  Database - CINAHL | 117,755 |
| S6 | TI ((obstetric n1 (care* or clinic*)) or antenatal or antepartum or “high-risk pregnancy clinic*” or (outpatient n1 (care or clinic*)) or (prenatal n1 (care or clinic*)) or (“public health* centre*” or “public health* center*”) or midwif* or midwive*) or AB ((obstetric n1 (care* or clinic*)) or antenatal or antepartum or “high-risk pregnancy clinic*” or (outpatient n1 (care or clinic*)) or (prenatal n1 (care or clinic*)) or (“public health* centre*” or “public health* center*”) or midwif* or midwive*) | Expanders - Apply equivalent subjects  Search modes - Boolean/Phrase | Interface - EBSCOhost Research Databases  Search Screen - Advanced Search  Database - CINAHL | 87,978 |
| S5 | (MH "Obstetrics") OR (MH "Delivery, Obstetric+") OR (MH "Obstetric Service") OR (MH "Obstetricians") OR (MH "Midwives+") OR (MH "Midwifery Service+") OR (MH "Prenatal Care") OR (MH "Perinatal Nurses") OR (MH "Perinatal Care") OR (MH "Perinatal Nursing+") OR (MH "Outpatient Service") OR (MH "Ambulatory Care Facilities+") | Expanders - Apply equivalent subjects  Search modes - Boolean/Phrase | Interface - EBSCOhost Research Databases  Search Screen - Advanced Search  Database - CINAHL | 94,217 |
| S4 | TI (qualitative or narrative or “semi-structured” or unstructured or informal or structured or interview* or questionnaire* or survey* or “focus group*” or ethnograph* or “field work” or naturalistic or phenomenology) or AB(qualitative or narrative or “semi-structured” or unstructured or informal or structured or interview* or questionnaire* or survey* or “focus group*” or ethnograph* or “field work” or naturalistic or phenomenology) | Expanders - Apply equivalent subjects  Search modes - Boolean/Phrase | Interface - EBSCOhost Research Databases  Search Screen - Advanced Search  Database - CINAHL | 927,496 |
| S3 | (MH "Interviews+") OR (MH "Unstructured Interview") OR (MH "Semi-Structured Interview") OR (MH "Structured Interview") OR (MH "Qualitative Studies+") OR (MH "Focus Groups") OR (MH "Ethnographic Research") OR (MH "Anthropology, Cultural") OR (MH "Surveys+") OR (MH "Questionnaires+") | Expanders - Apply equivalent subjects  Search modes - Boolean/Phrase | Interface - EBSCOhost Research Databases  Search Screen - Advanced Search  Database - CINAHL | 947,297 |
| S2 | TI (attitude* or view* or perception* or satisf* or experienc* or perceiv* or influenc* or facilitat* or barrier*) or AB (attitude* or view* or perception* or satisf* or experienc* or perceiv* or influenc* or facilitat* or barrier*) | Expanders - Apply equivalent subjects  Search modes - Boolean/Phrase | Interface - EBSCOhost Research Databases  Search Screen - Advanced Search  Database - CINAHL | 1,381,059 |
| S1 | (MH "Patient Satisfaction+") | Expanders - Apply equivalent subjects  Search modes - Boolean/Phrase | Interface - EBSCOhost Research Databases  Search Screen - Advanced Search  Database - CINAHL | 64,618 |

Bottom of Form

# PsycINFO

| **#** | **Query** | **Limiters/Expanders** | **Last Run Via** | **Results** |
| --- | --- | --- | --- | --- |
| S13 | S9 AND S10 AND S11 AND S12 | Expanders - Apply equivalent subjects Search modes - Boolean/Phrase | Interface - EBSCOhost Research Databases Search Screen - Basic Search Database - APA PsycInfo | 454 |
| S12 | S7 OR S8 | Expanders - Apply equivalent subjects Search modes - Boolean/Phrase | Interface - EBSCOhost Research Databases Search Screen - Basic Search Database - APA PsycInfo | 35,082 |
| S11 | S5 OR S6 | Expanders - Apply equivalent subjects Search modes - Boolean/Phrase | Interface - EBSCOhost Research Databases Search Screen - Basic Search Database - APA PsycInfo | 46,671 |
| S10 | S3 OR S4 | Expanders - Apply equivalent subjects Search modes - Boolean/Phrase | Interface - EBSCOhost Research Databases Search Screen - Basic Search Database - APA PsycInfo | 1,213,138 |
| S9 | S1 OR S2 | Expanders - Apply equivalent subjects Search modes - Boolean/Phrase | Interface - EBSCOhost Research Databases Search Screen - Basic Search Database - APA PsycInfo | 2,166,297 |
| S8 | TI ((“high risk*” n3 (pregnan* or gestation or birth or childbirth)) or ((gestation* or pregnan*) n2 diabet*) or “f?etal macrosomia*” or ((f?etal or f?etus or f?etous or embryo* or perinat* or neonat*) n2 (death* or mortality)) or stillborn or (“still born*” or “still birth*”) or ((birth or f?etal*) n2 (defect* or abnormal*)) or (“f?etal alcohol”) or FASD or FASDS or ((f?etal or f?etus or intrauterine) n1 (growth restrict* or restrict* growth*)) or ((f?etal or f?etus) n1 (hypoxi* or anoxi*)) or ((pregnan* or gestation*) n2 hypertensi*) or pre-eclampsia or eclampsia or “HELLP syndrome” or “hyperemesis gravidarum” or “pregnancy pernicious vomiting” or “abrupti* placenta*” or “premature rupture* f?etal membrane*” or “preterm prom” or (premature* n1 (labo?r or birth* or childbirth* or contraction*)) or ((increta or accreta or precreta or previa) n1 placenta*) or oligohydramnios or ((pregnan* or maternal) n1 (phenylketonuria or phenylalanine or pku)) or (placenta* n1 (insufficien* or fail*)) or (pregnan* n2 complicat*) or “adverse birth outcome*” or ((“cystic fibrosis” or lupus or “thyroid disease*” or “thyroid disorder*” or hyperthyroid* or hypothyroid*) n3 (pregnan* or childbirth* or labo?r*)) or (multiple n1 (pregnan* or gestation*)) or “multi f?etal gestation*” or twin* or triplet*) or AB ((“high risk*” n3 (pregnan* or gestation or birth or childbirth)) or ((gestation* or pregnan*) n2 diabet*) or “f?etal macrosomia*” or ((f?etal or f?etus or f?etous or embryo* or perinat* or neonat*) n2 (death* or mortality)) or stillborn or (“still born*” or “still birth*”) or ((birth or f?etal*) n2 (defect* or abnormal*)) or (“f?etal alcohol”) or FASD or FASDS or ((f?etal or f?etus or intrauterine) n1 (growth restrict* or restrict* growth*)) or ((f?etal or f?etus) n1 (hypoxi* or anoxi*)) or ((pregnan* or gestation*) n2 hypertensi*) or pre-eclampsia or eclampsia or “HELLP syndrome” or “hyperemesis gravidarum” or “pregnancy pernicious vomiting” or “abrupti* placenta*” or “premature rupture* f?etal membrane*” or “preterm prom” or (premature* n1 (labo?r or birth* or childbirth* or contraction*)) or ((increta or accreta or precreta or previa) n1 placenta*) or oligohydramnios or ((pregnan* or maternal) n1 (phenylketonuria or phenylalanine or pku)) or (placenta* n1 (insufficien* or fail*)) or (pregnan* n2 complicat*) or “adverse birth outcome*” or ((“cystic fibrosis” or lupus or “thyroid disease*” or “thyroid disorder*” or hyperthyroid* or hypothyroid*) n3 (pregnan* or childbirth* or labo?r*)) or (multiple n1 (pregnan* or gestation*)) or “multi f?etal gestation*” or twin* or triplet*) | Expanders - Apply equivalent subjects Search modes - Boolean/Phrase | Interface - EBSCOhost Research Databases Search Screen - Basic Search Database - APA PsycInfo | 27,351 |
| S7 | (((DE "Gestational Diabetes") OR (DE "Fetal Alcohol Syndrome")) OR (DE "Phenylketonuria")) OR (DE "Multiple Births" OR DE "Triplets" OR DE "Twins") or DE "Premature Birth" | Expanders - Apply equivalent subjects Search modes - Boolean/Phrase | Interface - EBSCOhost Research Databases Search Screen - Basic Search Database - APA PsycInfo | 17,085 |
| S6 | TI ((obstetric n1 (care* or clinic*)) or antenatal or antepartum or “high-risk pregnancy clinic*” or (outpatient n1 (care or clinic*)) or (prenatal n1 (care or clinic*)) or (“public health* centre*” or “public health* center*”) or midwif* or midwive*) or AB ((obstetric n1 (care* or clinic*)) or antenatal or antepartum or “high-risk pregnancy clinic*” or (outpatient n1 (care or clinic*)) or (prenatal n1 (care or clinic*)) or (“public health* centre*” or “public health* center*”) or midwif* or midwive*) | Expanders - Apply equivalent subjects Search modes - Boolean/Phrase | Interface - EBSCOhost Research Databases Search Screen - Basic Search Database - APA PsycInfo | 24,619 |
| S5 | (((((DE "Obstetrics" OR DE "Caesarean Birth" OR DE "Midwifery") OR (DE "Obstetricians")) OR (DE "Prenatal Care" OR DE "Childbirth Training")) OR (DE "Perinatal Period")) OR (DE "Outpatients")) OR (DE "Outpatient Treatment" OR DE "Outpatient Commitment") | Expanders - Apply equivalent subjects Search modes - Boolean/Phrase | Interface - EBSCOhost Research Databases Search Screen - Basic Search Database - APA PsycInfo | 30,209 |
| S4 | TI (qualitative or narrative or “semi-structured” or unstructured or informal or structured or interview* or questionnaire* or survey* or “focus group*” or ethnograph* or “field work” or naturalistic or phenomenology) or AB(qualitative or narrative or “semi-structured” or unstructured or informal or structured or interview* or questionnaire* or survey* or “focus group*” or ethnograph* or “field work” or naturalistic or phenomenology) | Expanders - Apply equivalent subjects Search modes - Boolean/Phrase | Interface - EBSCOhost Research Databases Search Screen - Basic Search Database - APA PsycInfo | 1,202,359 |
| S3 | ((((((DE "Interviews" OR DE "Cognitive Interview" OR DE "Focus Group Interview" OR DE "Intake Interview" OR DE "Interview Schedules" OR DE "Job Applicant Interviews" OR DE "Psychodiagnostic Interview" OR DE "Semi-Structured Interview") OR (DE "Qualitative Methods" OR DE "Focus Group" OR DE "Grounded Theory" OR DE "Interpretative Phenomenological Analysis" OR DE "Narrative Analysis" OR DE "Semi-Structured Interview" OR DE "Thematic Analysis")) OR (DE "Focus Group" OR DE "Focus Group Interview")) OR (DE "Ethnography")) OR (DE "Anthropology")) OR (DE "Surveys" OR DE "Consumer Surveys" OR DE "Mail Surveys" OR DE "Online Surveys" OR DE "Telephone Surveys")) OR (DE "Questionnaires" OR DE "General Health Questionnaire") | Expanders - Apply equivalent subjects Search modes - Boolean/Phrase | Interface - EBSCOhost Research Databases Search Screen - Basic Search Database - APA PsycInfo | 96,016 |
| S2 | TI (attitude* or view* or perception* or satisf* or experienc* or perceiv* or influenc* or facilitat* or barrier*) or AB (attitude* or view* or perception* or satisf* or experienc* or perceiv* or influenc* or facilitat* or barrier*) | Expanders - Apply equivalent subjects Search modes - Boolean/Phrase | Interface - EBSCOhost Research Databases Search Screen - Basic Search Database - APA PsycInfo | 2,166,018 |
| S1 | DE "Client Satisfaction" | Expanders - Apply equivalent subjects Search modes - Boolean/Phrase | Interface - EBSCOhost Research Databases Search Screen - Basic Search Database - APA PsycInfo | 6,541 |

Bottom of Form
